# Structural insights into the activation of ataxia-telangiectasia mutated by oxidative stress

**DOI:** 10.1126/sciadv.adi8291

**Published:** 2023-09-27

**Authors:** Anna C. Howes, Olga Perisic, Roger L. Williams

**Affiliations:** MRC Laboratory of Molecular Biology, Cambridge CB2 0QH, UK.

## Abstract

Ataxia-telangiectasia mutated (ATM) is a master kinase regulating DNA damage response that is activated by DNA double-strand breaks. However, ATM is also directly activated by reactive oxygen species, but how oxidative activation is achieved remains unknown. We determined the cryo-EM structure of an H_2_O_2_-activated ATM and showed that under oxidizing conditions, ATM formed an intramolecular disulfide bridge between two protomers that are rotated relative to each other when compared to the basal state. This rotation is accompanied by release of the substrate-blocking PRD region and twisting of the N-lobe relative to the C-lobe, which greatly optimizes catalysis. This active site remodeling enabled us to capture a substrate (p53) bound to the enzyme. This provides the first structural insights into how ATM is activated during oxidative stress.

## Teaser

Oxidative stress twists ATM dimers to remove a block to substrate binding and remodel the active site for optimal catalysis.

## INTRODUCTION

Ataxia-telangiectasia (A-T) is an autosomal recessive disorder that results from biallelic mutations in the A-T mutated (ATM) gene, causing ATM deficiency or production of nonfunctional ATM protein (*1*). The hallmarks of the disease are cerebellar dysfunction, premature aging, cancer predisposition, and immunodeficiency. ATM is a master regulator Ser/Thr protein kinase best known for signaling DNA damage and initiating cell cycle arrest, yet key A-T disease phenotypes such as neurodegeneration cannot be explained by ATM’s role in the DNA damage response alone. Elevation in free radical levels results in neuronal death (*2*, *3*), and it has long been hypothesized that neuronal aspects of A-T could result from increased oxidative stress (*4*, *5*). A number of studies have linked ATM to both sensing and controlling oxidative stress: ATM deficiency impairs mitochondrial response to oxidative stress (*6*–*9*), and reactive oxygen species (ROS) levels are substantially increased in ATM-deficient mice cerebella, mice thymocytes, human patient cells, and cell cultures (*2*, *3*, *7*–*10*). ATM is directly activated by oxidative stress in vitro and in cells (*8*, *10*). Upon activation by ROS, ATM regulates numerous downstream pathways such as cell cycle checkpoints (*8*, *11*), mitophagy (*9*, *12*), protein homeostasis (*8*), and ROS-dependent autophagy (*13*) and alters ROS levels by up-regulating pathways that generate the cellular reducing agent reduced form of nicotinamide adenine dinucleotide phosphate (NADPH) (*14*). An ATM mutant that responds to DNA damage but is unable to sense oxidative stress retains almost all the A-T phenotype in patients, suggesting that the inability of ATM to sense and signal oxidative damage has clinically relevant manifestations (*10*). Moreover, mutation of ATM may promote tumor formation through elevating ROS levels to up-regulate protumorigenic pathways (*15*). Overall, ATM emerges as a key cellular sensor of oxidative stress, initiating a signaling cascade to respond to and alleviate deleterious oxidative damage. However, the molecular mechanism of how ATM senses and responds to oxidative damage remains unknown, which hinders the development of targeted medicinal therapies. To understand how oxidative stress activates ATM, we determined a cryo–electron microscopy (cryo-EM) structure of ATM activated by H_2_O_2_ bound to a p53 substrate peptide, enabling us to propose a model for redox activation and test this model using biochemical and kinetic methods.

ATM belongs to the phosphatidylinositol-3 kinase–related kinase (PIKK) superfamily, whose members share a common domain architecture. The N terminus consists of an α-helical solenoid that is not homologous among members of the family, varies in length, and mediates protein-protein interactions (*16*, *17*). The C-terminal region contains the conserved FRAP-ATM-TRRAP (FAT) and kinase domains (collectively known as the FATKIN), with a FAT-C–terminal (FATC) domain at the extreme C terminus (fig. S1). Cryo-EM structures of the human and *Schizosaccharomyces pombe* ATM (*18*–*23*) and *Saccharomyces cerevisiae* and *Chaetomium thermophilum* Tel1 proteins (*24*–*27*) showed two 350-kDa monomers forming a dimer via FATKIN elements. The available structures captured an ATM dimer with signature features, suggesting that it is only basally active, with the kα9b helix of the autoinhibitory PIKK regulatory domain (PRD) element occupying the peptide substrate binding site and with the relationship of the kinase N-lobe relative to the C-lobe most closely resembling the structure of mammalian target of rapamycin complex 1 (mTORC1) in its basal state (*21*, *28*), even in the presence of bound nucleotide (*21*, *24*, *27*).

During the DNA damage response, it was postulated that the Mre11-Rad50-Nbs1 (MRN) complex senses the double-stranded DNA (dsDNA) break and directly activates ATM by promoting a dimer-to-monomer transition (*29*, *30*). However, ATM activation by ROS appears to promote disulfide bond formation between ATM dimer–related protomers via C2991 residues (*8*, *10*). Our structural results suggest how disulfide bond formation between ATM protomers promotes large-scale rearrangement of the kinase domain necessary for activation.

## RESULTS

### H_2_O_2_ activates ATM in vitro

**Fig. 1.**
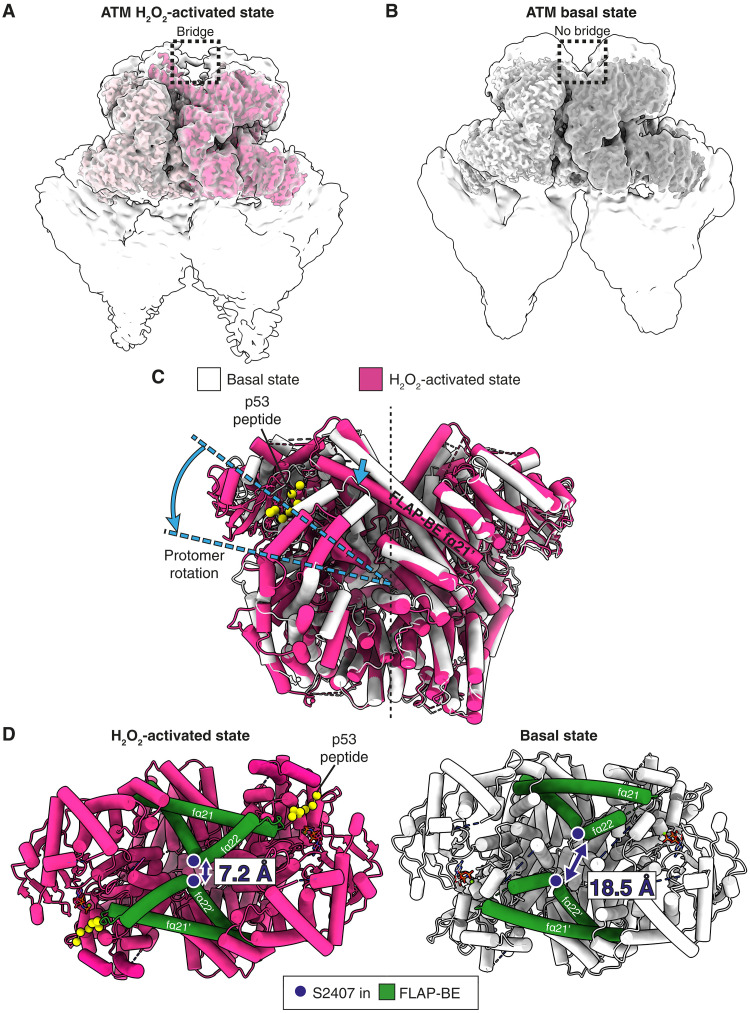
Cryo-EM maps and models for the ATM H_2_O_2_-activated and basal states. (**A**) ATM H_2_O_2_-activated state cryo-EM map for a locally refined dimeric C-terminal region that includes the FATKIN (light and deep pink), which was resolved to 3.0 Å, superimposed on the map for the whole dimer (translucent). The dimer consensus map was low-pass-filtered to 7 Å to visualize the low-resolution, flexible bridge density connecting the protomers of the dimeric head. (**B**) ATM basal state cryo-EM maps superimposed as in (A). The dimer consensus map has been low-pass-filtered to 7 Å in the same manner as the H_2_O_2_-activated state map, and no additional bridge density is present. (**C**) The FATKINs of the H_2_O_2_-activated and basal states are superimposed by aligning on the right protomer. Large conformational changes are denoted with light blue arrows. (**D**) Top views of the ATM H_2_O_2_-activated state (left) and basal state (right) FATKIN models. Large changes in conformation are emphasized by highlighting distances between the dimer-related S2407 residue pair.

To understand the mechanism of redox activation of ATM, we assayed purified, recombinant human ATM to optimize conditions for cryo-EM structures. We used Phos-Tag gels and an N-terminal p53 peptide (p53^1–102^) as a model substrate for quantitative substrate phosphorylation assays (*31*). This ATM could be activated by either MRN in the presence of DNA or by oxidative stress induced using H_2_O_2_, consistent with previous reports (fig. S2A) (*10*, *30*, *32*). Phosphorylation did not occur with a kinase-dead ATM mutant or with ATM inhibitor, confirming that the shifts observed in the assay are due to ATM-dependent substrate phosphorylation (fig. S2A). Consistent with previous results (*10*), mutating C2991 to either Leu or Ser abolished H_2_O_2_-based ATM activation (fig. S2B). We also showed that our recombinant ATM directly phosphorylated checkpoint kinase 2 (CHK2) and nuclear respiratory factor 1 (NRF1; fig. S2, C and D), two other previously established redox-sensitive targets (*7*, *10*, *33*). We identified an ATM point mutant, Q2971A, which showed greater basal and H_2_O_2_-activated substrate phosphorylation (fig. S2B). This residue is part of the autoinhibitory PRD that was proposed to inhibit ATM by mimicking substrate binding based on substrate interactions observed for the nonsense mediated mRNA decay associated PIKK SMG1 (*34*). We incorporated this mutant in our structural analysis so that we could determine how ATM recognizes its substrate. To determine which H_2_O_2_ concentration to use for ATM activation, we assayed initial ATM activity as a function of H_2_O_2_ concentration (fig. S3). However, the substrate response curve had neither a sigmoidal nor hyperbolic shape (fig. S3). Instead, activity increased to a peak followed by decrease at higher H_2_O_2_ concentrations. Speculating that the decrease at higher H_2_O_2_ concentration may be due to disulfide link–induced aggregation, we prepared a sample for cryo-EM structural analysis using conditions under which the Q2971A ATM was maximally activated by H_2_O_2_ (fig. S4). We observed two conformational states of ATM in this sample: (i) an H_2_O_2_-activated conformation of ATM with bound p53 peptide substrate (p53^11–22^) resolved to 3.0 Å for the C-terminal head of the ATM dimer (Fig. 1A, pink), with an extra low-level density bridging the two kinase domains in the dimer in the consensus map (Fig. 1A, translucent) and (ii) a 2.5-Å resolution ATM dimer in its basal state with no p53 peptide substrate bound and with a conformation similar to previously published inactive ATM structures (Fig. 1B and figs. S5 and S6) (*20*, *21*). H_2_O_2_-activated state particles represent only a small population of the dimers that we observe from cryo-EM analysis. This may be because of the low H_2_O_2_ concentration that we had to use to limit aggregation. Most of the dimers present in the cryo-EM sample have a basal state conformation.

### H_2_O_2_ activates ATM by forming twisted dimers that eject the PRD

H_2_O_2_-activated ATM exists as a dimer (Fig. 1A). During our data analysis, we did not observe ATM monomers, consistent with results suggesting that H_2_O_2_ activation involves disulfide bond formation between ATM protomers (*10*). The activated conformation of ATM shows a substantial rearrangement of the FATKIN domains compared to the basal state (Fig. 1C). By superimposing one protomer of the basal dimer on the corresponding protomer of the activated dimer, it is apparent that the other protomer is rotated relative to the aligned protomer (Fig. 1C and movie S1). This conformational change is accompanied by notable changes at ATM’s dimeric interface, which will be described below in more detail. The major movement involves helices fα21 and fα22 (residues 2377 to 2476), referred to as the FLAP-BE [FLAP-binding element, where FLAP stands for FATC, LBE (kα4b-kα4c, residues 2793 to 2828), activation loop, and PRD]. Upon H_2_O_2_ activation, the fα21 helix of the FLAP-BE tilts downward (Fig. 1C) and shifts inward (Fig. 1D), while the fα22 helix kinks inward to form elbows centered on residues S2407 that nearly touch each other (Fig. 1D). In the consensus map for H_2_O_2_-activated ATM, we observe additional density that crosses the approximate twofold axis between ATM protomers (Fig. 1A and movie S2). This bridging density is absent in both our and other previously published basal ATM structures (Fig. 1B), and we attribute it to a disulfide bridge between the two protomers formed under oxidizing conditions.

**Fig. 2.**
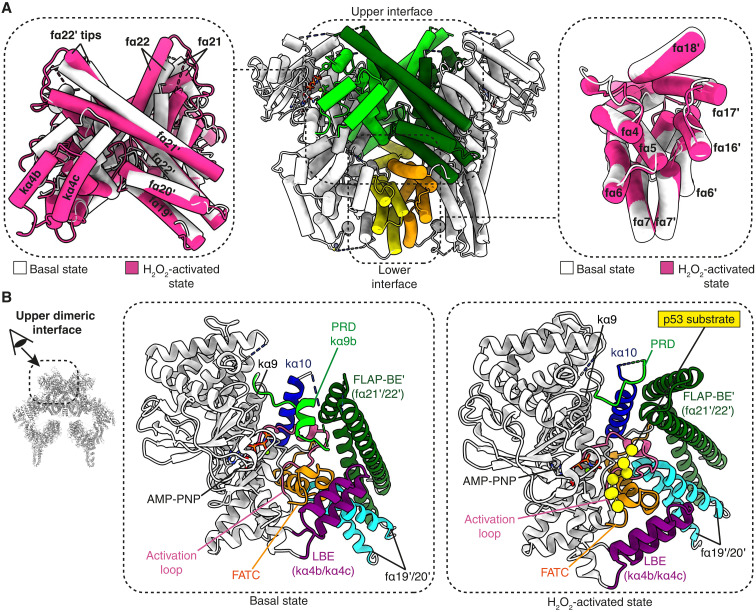
Changes occur at the upper dimeric interface between the basal state and H_2_O_2_-activated state. (**A**) The upper (light and dark green) and lower (yellow and orange) dimeric interfaces of ATM are highlighted for the basal state (middle). The superimposed basal and H_2_O_2_-activated states are shown for each interface (left and right). The lower interface shows little change between both states (right), whereas there are drastic changes in the upper interface (left). (**B**) The conformational differences between states change the relationships between key elements that make contact at the dimeric interface, as shown by juxtaposing the basal and H_2_O_2_-activated states. Only interactions of FLAP-BE (fα21-fα22) and fα19/fα20 from one protomer with elements from the opposing protomer are shown.

Basal ATM forms a dimer through two key interfaces in the C-terminal half of the protein (Fig. 2A and movie S3). The lower interface involves interactions between tetratricopeptide repeat domain 2 (TRD2) fα5-fα7 helices and TRD3 fα16-fα18 helices of each protomer (Fig. 2A, yellow and orange). These interactions remain intact in the H_2_O_2_-activated state. The upper interface (Fig. 2A, light and dark green) involves the FATC, LBE, activation loop (residues 2888 to 2910), kα10 (residues 3001 to 3018), and PRD (residues 2954 to 3000) of one protomer interacting with FLAP-BE′ of the other protomer (Fig. 2B). Notable differences can be seen in the upper interface when comparing the H_2_O_2_-activated state to the basal state (movie S3). Interactions between the PRD and FLAP-BE′ helices are drastically altered as the PRD path completely changes in the activated state compared to the basal state: Instead of the PRD occupying the substrate binding site, as it does in the basal state, the PRD moves behind the FLAP-BE′ helices in the activated state (Fig. 2B, right, light green). Contacts between the FLAP-BE′ of one protomer and LBE of the other protomer are completely lost in the H_2_O_2_-activated state (Fig. 2B, dark green and purple). The remaining interfaces are also altered, including contacts of C-lobe catalytic elements (activation loop, kα10, and the kα10/FATC loop) with the FLAP-BE′ from the paired protomer. In addition, the FATC changes its interactions with the fα19′/fα20′ loop and kα10′ from the paired protomer (table S2). The combined protomer rotation and PRD displacement exposes the substrate binding site, with residues from the LBE, catalytic loop, activation loop, and FATC becoming freely available to interact with the p53 substrate peptide (Fig. 2B, right).

### p53 substrate is bound to the H_2_O_2_-activated ATM

**Fig. 3.**
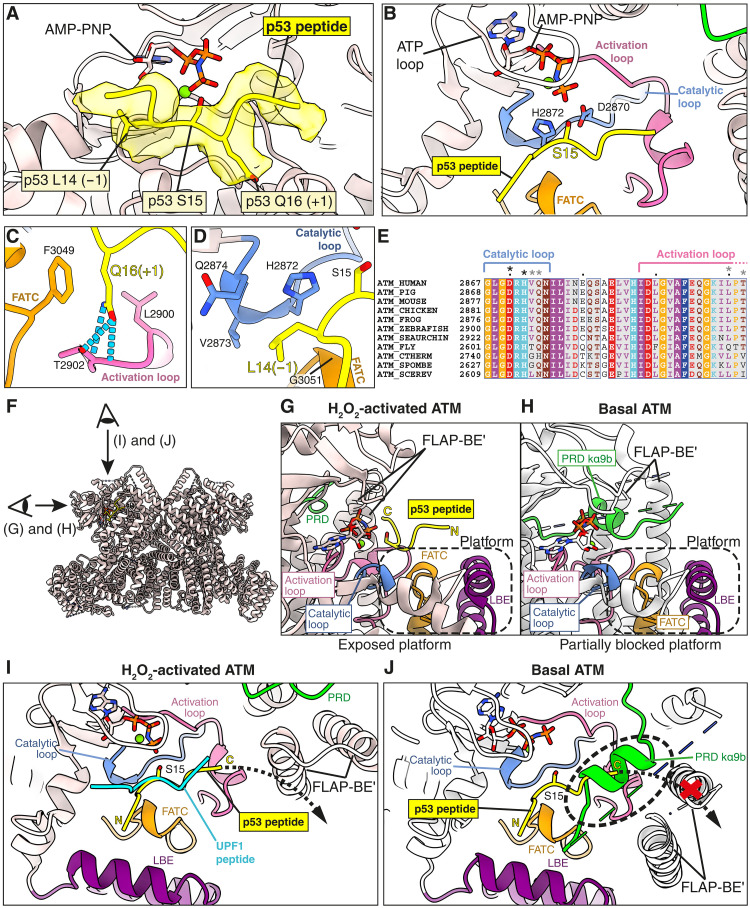
Binding of p53 substrate peptide in the ATM active site. (**A**) Additional density that corresponds to the p53 peptide is observed in the H_2_O_2_-activated state cryo-EM map, which corresponds to p53 residues 12 to 18. (**B**) Recognition of p53 Ser^15^, the phospho-acceptor residue. (**C**) Recognition of p53 Gln^16^ (position +1) by ATM. (**D**) Recognition of p53 Leu^14^ (position −1) by ATM. (**E**) Residues in the catalytic and activation loops that are involved in interactions with the p53 substrate peptide are highlighted above the aligned ATM sequences from several species. (**F**) Illustration of views that are used for (G) to (J). (**G**) A platform for substrate binding is fully exposed in the H_2_O_2_-activated state, whereas in (**H**), the platform is blocked by the PRD kα9b helix and the FLAP-BE of the other protomer. (**I**) The path of a potential continuation for p53 is highlighted in the H_2_O_2_-activated state structure (dashed black arrow), and the UPF1 peptide taken from the structure of the peptide bound to the PIKK SMG1 (PDB: 6Z3R, superimposed using the kinase C-lobe). (**J**) Substrate binding is blocked in the basal state ATM structure.

There was no density for substrate peptide in the basal state particles in the H_2_O_2_-treated sample, but in the population of the activated ATM dimers (in the same sample), we observed additional density in the active site for the 12-residue p53 peptide (p53^11–22^) (Fig. 3A). Seven of the twelve p53 peptide residues (12-PPLSQET-18) were visible. The phospho-acceptor site in the peptide is a serine (Ser^15^ of the full-length p53 sequence), and the hydroxyl group is positioned by Asp^2870^ and His^2872^ from the catalytic loop (Fig. 3B). The residues flanking the acceptor serine interact with the catalytic loop, activation loop, and FATC. The p53 Gln^16^ (position +1) appears to form hydrogen bonds with the peptide backbone and side chain of catalytic loop Thr^2902^ and hydrophobic interactions with Leu^2900^ and Phe^3049^ (Fig. 3C). The p53 Leu^14^ (position −1) makes hydrophobic interactions with His^2872^, Val^2873^, and Gln^2874^ of the catalytic loop and Gly^3051^ of FATC (Fig. 3D). Langer *et al.* (*34*) observed similar recognition sites for the SMG1-8-9 kinase with upstream frameshift 1 (UPF1) peptide (Fig. 3I). ATM residues interacting with the S/Q motif of the substrate are highly conserved (Fig. 3E), consistent with the selectivity of ATM for this motif. Two of the residues interacting with the −1 position of the substrate are less conserved, which is consistent with the greater range of substrate residues tolerated at this position (*35*–*37*).

In the H_2_O_2_-activated state, a platform consisting of the LBE, catalytic loop, activation loop, and FATC is exposed, on which the substrate binds and presents its phospho-acceptor site to the adjacent adenylyl-imidodiphosphate (AMP-PNP; Fig. 3G). In the basal state, the site occupied by the substrate positions +1 to +3 in the active state is blocked by PRD helix kα9b (Fig. 3H). In addition, in the basal state, the FLAP-BE′ from the other protomer forms a wall, which retreats to the back of the platform when the FLAP-BE′ tilts in the active state (Fig. 3, I and J). This could create a less restrictive space to accommodate a longer substrate peptide.

### Twisted ATM dimers shift the kinase N-lobes to optimize catalysis

**Fig. 4.**
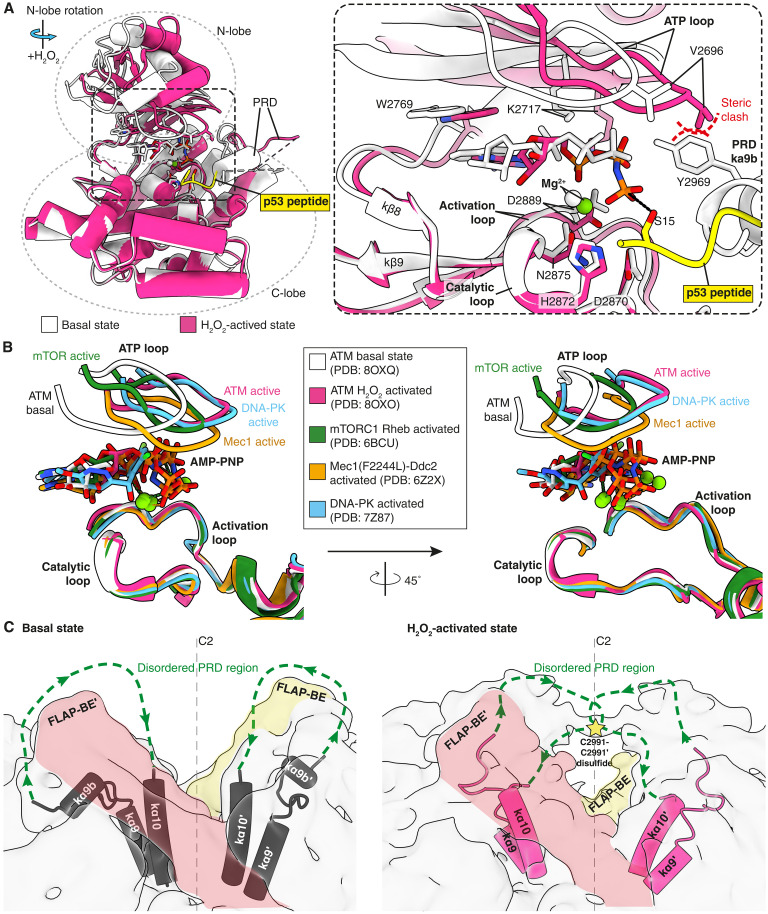
The H_2_O_2_-activated state displaces the PRD, repositions C2991 for disulfide bond formation, and remodels the ATP-binding site. (**A**) Kinase domains for the basal state and H_2_O_2_-activated state are superimposed and aligned on the kinase C-lobe (left). The N-lobe is rotated in the H_2_O_2_-activated state, which repositions the nucleotide phosphates and magnesium ion closer to residues in the catalytic loop and the phospho-acceptor Ser^15^ (right inset). (**B**) Activated PIKK structures are superimposed on the ATM basal structure, aligning on the catalytic loop and activation loop in the kinase C-lobe, to compare the ATP loop shifts in the N-lobe. Two views are shown and are rotated 45° relative to one another. (**C**) Plausible paths for the disordered PRDs are illustrated as dashed green lines with arrows indicating the N- to C-terminal direction for the basal state (left) and H_2_O_2_-activated state (right). FLAP-BE densities are colored (orange and yellow) to indicate the density in the basal state that the PRD must circumvent. In contrast, the FLAP-BE is not an obstacle for the H_2_O_2_-activated state PRD because the PRD has moved closer to the dimer symmetry axis.

To compare changes occurring within the active site, protomers for the H_2_O_2_-activated and basal states were superimposed on the kinase domain C-lobe. Within each protomer, there are two major hinges in the structure that are involved in the transition from the basal to the activated state. One hinge is near the junction of TRD1 with TRD2 (around residue 2022), and it results in TRD2, TRD3, huntingtin, elongation factor 3, protein phosphatase 2A and TOR (HEAT) repeat domain (HRD), and the N-lobe of the kinase domain moving as a unit with respect to the C-lobe of the kinase domain. The other hinge is in the link between the N- and C-lobes of the kinase domain (around residue 2773) and enables a distinct rotation of the N-lobe relative to the C-lobe, going from the basal state to the H_2_O_2_-activated state (Fig. 4A and movie S4). This N-lobe movement shifts the adenosine triphosphate (ATP) loop (kβ3/kβ4 loop, residues 2693 to 2699) relative to the activation loop in the C-lobe (movie S5). This movement only appears possible after PRD kα9b has been removed from the substrate binding site; otherwise, V2696 in the ATP loop would clash with Y2969 of the PRD kα9b helix in the basal state (Fig. 4A, right). N-lobe rotation shifts K2717, which makes direct contact with the ATP α-phosphate group, as well as W2769, which interacts via aromatic-stacking with the adenine base. These shifts bring ATP closer to both the 2970-DRH-2972 motif in the catalytic loop and the phospho-acceptor Ser^15^ in the p53 substrate. The conformational change of the nucleotide in the H_2_O_2_-activated state also positions the γ-phosphate closer to p53 Ser^15^ (Fig. 4A, right). Similar changes in the relationship of the N-lobe to the C-lobe and associated ATP loop shifts were observed for other PIKK family members upon activation, such as in Ras homolog enriched in brain (RHEB)– activated mTORC1 (*28*), constitutively active Mec1(F2244L) complex (*38*), and activated DNA-dependent protein kinase (Fig. 4B and fig. S7) (*39*, *40*). PIKK structures were aligned on the activation loop and catalytic loop, which show high sequence conservation (fig. S8).

### The twisted ATM dimer facilitates the formation of a critical disulfide bond

A key consequence of PRD displacement from the substrate binding site is that C2991 residues can be brought into close enough proximity to form an interprotomer disulfide bond under oxidizing conditions. This would be consistent with the bridge density that we observed in the consensus map (Fig. 1A), and a plausible position for the disulfide bond within this bridge is illustrated [Fig. 4C (right) and fig. S9A]. High-resolution density is missing for a large portion of the PRD in the basal and H_2_O_2_-activated structures, as this remains a highly flexible region. To compare this region of the PRDs between basal and H_2_O_2_-activated states, we have illustrated plausible paths based on the direction in which the density for the PRD continues when each structure is viewed at low-density thresholds (Fig. 4C and fig. S9). In the basal state, the PRD path after kα9b points away from the twofold axis of the dimer so that it would need to bend back to get around the FLAP-BE and reach kα10 [Fig. 4C (left) and fig. S9B]. This would restrict the ability of C2991 residues to come within proximity to one another at the twofold axis of the dimer and prevent disulfide bond formation. In contrast, the PRD path in the H_2_O_2_-activated state sits behind the FLAP-BE helices (Fig. 2B and fig. S9A) and already extends toward the twofold dimer axis (Fig. 4C, right), bringing it much closer to kα10 to which it must connect, making disulfide bond formation between C2991 residues much more feasible [Fig. 4C (right) and fig. S9A].

### Structural observations enable proposal of a model of ATM activation by oxidative stress

**Fig. 5.**
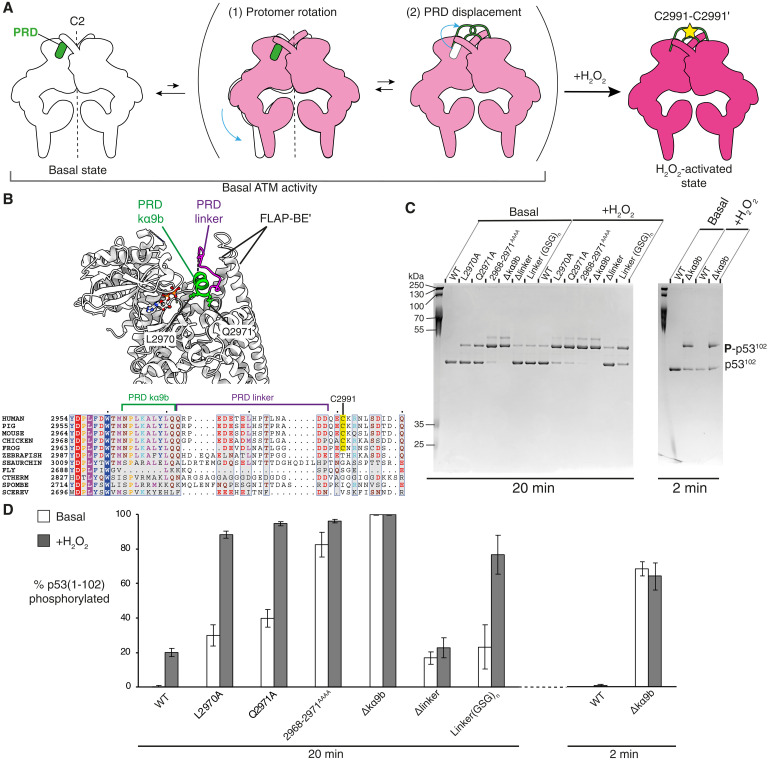
A model for ATM activation by H_2_O_2_, with validation by structure-guided mutagenesis. (**A**) Cartoons represent the ATM dimer in basal and H_2_O_2_-activated states. Under basal conditions, ATM basal state is proposed to be in equilibrium with a rotated, PRD-displaced conformation, with the equilibrium greatly favoring the basal state. We propose that H_2_O_2_ captures the rotated, PRD-displaced conformation through C2991 disulfide bond formation. (**B**) A model of ATM focusing on the kinase domain to highlight key features and residues that were mutated for kinase assays. (**C**) A representative Coomassie-stained Phos-Tag gel from ATM mutant kinase assays. Final concentrations of components (where used): 25 nM ATM, 250 nM MRN, 10 nM DNA, 0.5 mM H_2_O_2_, 5 μM MBP-p53(1-102), 1 mM ATP, and 5 mM MgCl_2_. The intensities of the phosphorylated and nonphosphorylated bands were quantitated by scanning Coomassie-stained gels to determine the percent phosphorylation of each sample. (**D**) Bar chart showing the average percent substrate phosphorylation and SD for each ATM construct as observed from Phos-Tag gels using three biological replicates.

On the basis of our structural observations, it is apparent that large conformational changes are occurring both between two protomers within a dimer and within each protomer during the transition from the basal state to the H_2_O_2_-activated state. Although ATM has basal activity in the absence of any activators (fig. S10A) (*21*), structures determined in the absence of an activator have features indicative of an inactive conformation (*20*, *27*), which are nearly identical to our basal state conformation. It is possible that ATM under basal conditions can also transition, albeit infrequently, between the basal state and an activated state (Fig. 5A) that is similar to the state that we observed for the H_2_O_2_-activated ATM. Alternatively, in the absence of H_2_O_2_, it may be that the activated state is not identical to the H_2_O_2_-activated state of ATM. Regardless of conformational change responsible for basal activity, it is likely to involve a kinetically unfavorable transition so that only a very small fraction of the population would adopt this state, making it structurally inaccessible.

Whether protomer rotation and PRD displacement during activation occur simultaneously or in a stepwise manner (Fig. 5A) is unknown. The displacement of the PRD results in C2991 residues being brought into close enough proximity for an interprotomer disulfide bond to form (Figs. 4C and 5A). Upon addition of H_2_O_2_, a disulfide bond can form between C2991 residues from each ATM protomer so that the PRD can be stabilized in a conformation that clears the substrate binding site (Fig. 3, G and I). This change could not only allow substrate to bind but also facilitate the rotated protomer state that we observe in our H_2_O_2_-activated structure, thereby optimizing the orientation of the N-lobe relative to the C-lobe for phosphate transfer to the peptide substrate.

### Structure-guided mutagenesis supports the proposed model of activation

Two predictions arise from our proposed model that we tested using structure-guided mutagenesis in the ATM active site (Fig. 5B). These mutants were then tested in Phos-Tag gel kinase assays, and then, the percentage of phosphorylated substrate was quantified (Fig. 5, C and D). First, we predicted that mutating residues that stabilize the PRD in the substrate binding site should result in an increase in ATM basal activity, and the addition of H_2_O_2_ should further activate ATM as the disulfide bridge captures the activated state. Two point mutations were generated, L2970A and Q2971A, in the PRD kα9b helix to test this idea (Fig. 5, C and D, and fig. S11). Both L2970A and Q2971A increased the basal activity of ATM. This is expected because L2970 and Q2971 side chains make interactions with the active site that would be lost in the alanine mutants, facilitating displacement of the PRD from its inhibitory position. Both mutants were further stimulated by H_2_O_2_, consistent with our model that H_2_O_2_ captures an active ATM state via disulfide bridge formation. Extending this by mutating four PRD residues to alanine (2968 to 2971^AAAA^) showed an even greater increase in basal activity, which could be further activated by H_2_O_2_. Completely deleting the PRD kα9b helix (Δkα9b, residues 2963 to 2972) led to a substantial increase in substrate phosphorylation by ATM on its own. Consistent with a key step of ATM activation by H_2_O_2_ being removal of the autoinhibitory PRD kα9b element, Δkα9b could not be additionally stimulated by H_2_O_2_, although residue C2991 is still present in the construct. Deletion of the autoinhibitory kα9b helix likely disrupts interactions with FLAP-BE from the other protomer, thereby facilitating protomer rotation within the ATM dimer and remodeling of the active site.

Second, we predicted that mutants shortening the PRD linker segment between kα9b and C2991 would sterically prevent formation of the disulfide bond and prevent activation by H_2_O_2_. To this end, residues in the disordered linker were deleted (Δlinker, residues 2973 to 2988) to shorten the PRD region and prevent interprotomer disulfide bond formation. This construct shows an increase in basal activity compared to wild-type ATM that could be due to the disruption of potential autoinhibitory interactions between the PRD linker and the FLAP-BE′ that were observed at low-density thresholds in the basal state (fig. S12C). This additional PRD linker density was also observed in a previously published structure of human ATM bound to ATPγS (*20*). Notably, there is no further increase in activity with H_2_O_2_, presumably because the truncated PRD linker is too short to establish an interprotomer disulfide bond. Restoring the length of the PRD using a repetitive GSG sequence [linker (GSG)_4_GS, residues 2975 to 2988] restored activation by H_2_O_2_. As with the linker deletion, the basal activity is elevated compared to wild-type ATM, likely because specific interactions remain disrupted between the PRD linker and FLAP-BE′.

### Comparing kinetics of ATM activation by oxidative stress and by DNA damage

To understand how ATM activation by H_2_O_2_ compares to ATM activation in response to DNA damage, we determined Michaelis-Menten parameters for the basal, H_2_O_2_, and MRN/DNA-activated states using p53 as a substrate. For basal conditions, the catalytic turnover (*k*_cat_) was ~0.015 s^−1^, and the Michaelis constant (*K*_M_) was ~80 μM (fig. S10A). The H_2_O_2_ concentration that gave the maximum substrate phosphorylation rate was used to examine the kinetics of ATM activation by H_2_O_2_ (fig. S3). The *k*_cat_ increased to ~0.4 s^−1^, consistent with our observed rearrangements in the active site (Fig. 4A) that would promote faster substrate turnover, while *K*_M_ decreased to 29 μM, consistent with a greater availability of the substrate binding site promoted by PRD remodeling. In the presence of MRN and 350–base pair dsDNA, the *K*_M_ decreased to ~24 μM, suggesting that the substrate binding site is also made more accessible by MRN and DNA, perhaps also by displacement of the PRD. Activation by MRN/DNA increased *k*_cat_ to 12 s^−1^, which is substantially greater than the 0.4 s^−1^ produced by H_2_O_2_ activation. The lower fold of maximal activation produced by H_2_O_2_ (~30-fold) compared with the maximal fold activation elicited by MRN/DNA (~800-fold) suggests either a very different mechanism of activation for ATM in the presence of MRN/DNA (*8*) or that H_2_O_2_ is activating a smaller population of ATM molecules than MRN/DNA. If the mechanism of activation were entirely due to loss of autoinhibition by the PRD, we might expect that removing the PRD would lead to maximal activation. Consistent with this, we found that for a PRD Δkα9b mutant, the *k*_cat_ for basal activity increased to 13 s^−1^, similar to the *k*_cat_ for ATM activated by MRN/DNA. Furthermore, Δkα9b is not activated by either MRN/DNA or H_2_O_2_ (fig. S10B). Because the same maximal activity is achieved by both the Δkα9b mutant and MRN/DNA, it suggests that the lower maximal activity achieved in the presence of H_2_O_2_ arises because only a small portion of the population of ATM dimers are disulfide linked under the conditions of the assay, which agrees with our cryo-EM work. These results suggest that activation by H_2_O_2_ and MRN/DNA might share a common step, involving extraction of the PRD from the substrate-binding site despite them using, overall, very different mechanisms to achieve this.

In addition to measuring ATM protein kinase activity, we assessed its adenosine triphosphatase activity by measuring adenosine diphosphate (ADP) production in the absence of peptide substrate using an ADP-Glo kinase assay (fig. S13). The ATP hydrolysis by ATM in the basal state in the absence of p53 substrate is low; however, the addition of H_2_O_2_ increased the ATP hydrolysis by about fivefold. This suggests that the activation by H_2_O_2_ remodels the active site even in the absence of peptide substrate. Furthermore, ATM Δkα9b shows a great increase in ATP hydrolysis in the absence of peptide substrate, consistent with our proposal that removal of the PRD causes remodeling of the active site by realigning the N-lobe relative to the C-lobe.

## DISCUSSION

The structure of H_2_O_2_-activated ATM reveals a disulfide-stabilized ATM dimer, having a unique orientation of one protomer with respect to the other, with the autoinhibitory PRD element displaced and a p53 substrate peptide bound. Our structure-guided mutagenesis suggests that a key feature preventing disulfide stabilization in the basal dimer is that the PRD loop bearing C2991 is not well positioned for this residue to reach its counterpart in the dimer-related protomer. Once protomers are rotated and the PRD is displaced, the disulfide bond can form under oxidizing conditions to stabilize the enzyme in an activated conformation. This stabilized H_2_O_2_-activated state has a conformation that enables p53 peptide substrate binding and remodels the ATP-binding site to increase the catalytic rate. This mechanism of activation is unique for ATM relative to redox activation described for other kinases (*41*, *42*). The H_2_O_2_-activated state that we observe results from several structural changes, but the order of these changes is unknown. In addition to the critical cysteine modification at C2991, other oxidative modifications have been previously reported (*10*). These could collectively contribute to redox-mediated changes in ATM conformation, although on their own, they do not activate ATM (*10*).

Further insights could be gained into how ATM regulates the cell’s response to oxidative stress by identifying critical direct targets of ATM activated by oxidative stress. Phosphoproteomics studies identified numerous downstream phosphorylated peptides that are dependent on ATM activation by oxidative stress (*8*, *43*), although the direct targets of ATM phosphorylation need further validation in vitro with purified proteins. Loss of oxidation-induced ATM activation results in profound cellular deficiencies, the most prominent one being an increase in intracellular ROS, which is linked to mitochondrial dysfunction and defects in autophagy, proteostasis, and the antioxidant capacity of the pentose-phosphate pathway [reviewed in (*44*)]. For some of the direct targets of ROS-activated ATM, such as p53, CHK2, and NRF1, there is an established link to mitochondrial function (*7*, *10*, *33*); however, for many targets, a mechanistic link between phosphorylation by ATM and specific cellular consequences is missing.

The H_2_O_2_-activated state of ATM is likely to occur in any cellular location under oxidative stress. In contrast, ATM activation by MRN/DNA occurs at a DNA damage locus, followed by phosphorylation spread by one-dimensional (1D) unidirectional sliding of the enzyme along the chromatin away from the site of damage (*45*). Although ATM activation under DNA damage conditions and oxidizing conditions could share a common step of PRD removal from the substrate-binding site, the mechanism by which this is achieved by MRN/DNA is entirely different, as it involves a monomer-to-dimer transition (*29*, *32*), whereas activation by H_2_O_2_ involves the formation of a disulfide-bridged ATM dimer. This difference in oligomeric state might explain the activation behavior of the R3047X mutant of ATM, which lacks the last 10 C-terminal residues in the FATC domain and was shown to be activated by MRN/DNA but not by H_2_O_2_ (*10*). The pathway-specific activation of this mutant is quite intriguing because deletion of the FATC C terminus would disrupt interactions of the FATC with the activation loop, catalytic loop, LBE, and p53 substrate peptide (fig. S14), generally destabilizing the active site. It is possible that MRN and DNA interactions with an ATM monomer can stabilize the active site elements in the absence of the FATC C terminus to allow the activation of ATM, while in the H_2_O_2_-oxidized dimer, there are no compensatory interactions to stabilize the active sites. Unfortunately, the structural basis for the differential, pathway-specific effects of deletion of this important structural element remains speculative until the MRN/DNA-activated structure of ATM becomes available.

From our comparison of kinetic properties of ATM activated under different conditions (by MRN/DNA or by H_2_O_2_) and from our cryo-EM analysis of ATM activated by H_2_O_2_, we suggest that only a fraction of the population of molecules is being modified and activated by H_2_O_2_. However, the possibility remains that the differences in kinetic parameters are due to intrinsic, mechanistic differences between the two activated states or that perhaps both population and mechanistic effects could play roles in the kinetic differences that we observe. Development of more sophisticated single-molecule techniques, perhaps such as fluorescence resonance energy transfer–based techniques, might help address these questions. Furthermore, within a cellular context, additional confounding factors might be affecting the potency of ATM activation by different cellular stresses; therefore, further studies will be necessary to quantify the activation of ATM in cells.

ATM has long been known as a major tumor suppressor gene, and for more than a decade now, it has been established as a redox sensor in cells. The inability to effectively regulate ROS has clinically important consequences, as observed not only in the hallmarks of the ataxia-telangiectasia disorder but also potentially in cancer development. Our structural and biochemical data provide detailed molecular insights into ATM activation and substrate binding under oxidative stress conditions.

## MATERIALS AND METHODS

### Cloning of recombinant human ATM wild-type and mutant constructs

Expression of all ATM constructs was carried out using a pDEST12.2-OriP–ATM plasmid, which encodes an N-terminal FLAG sequence, FKBP1A (UniProt accession code P62942) followed by a nine–amino acid linker and the full-length human ATM sequence (1 to 3056; UniProt accession code Q13315) codon-optimized for human expression [described in (*18*)].

Cloning of all mutants was carried out using a fragment assembly–based approach: Two fragments were polymerase chain reaction (PCR)–amplified with overlaps containing either the desired mutant or deletion at one end and overlaps with the Bam HI/Xho I–digested FKBP-ATM plasmid at the other, with the combined fragments covering the entire ATM sequence. These fragments were assembled with the Bam HI/Xho I–digested FKBP-ATM plasmid using HiFi DNA Assembly.

### Expression and purification of recombinant human ATM

For the wild-type and all mutants of ATM, purification steps were followed as in (*18*) with modifications. Briefly, mammalian Expi293F cells were grown in Expi293 Expression Medium to a density of 2.5 × 10^6^ cells/ml at 37°C and 8% CO_2_ with 125-rpm shaking, transfected with DNA at 1.1 mg/liter cells and polyethylenimine (PEI) at 3 mg/liter cells, and incubated for 48 hours. Cells were then harvested at 3000*g* for 15 min at 4°C, flash-frozen in liquid nitrogen, and stored at −80°C until purification.

A cell pellet from 2 liters of culture was resuspended in 150 ml of cold lysis buffer [50 mM Hepes (pH 7.5 at 23°C), 150 mM NaCl, 100 mM arginine mono-HCl, and 10% glycerol (v/v), supplemented with EDTA-free protease cocktail inhibitor tablets (Roche, 05056489001), Universal Nuclease (Thermo Fisher Scientific, 88702), and Pefabloc (Melford, A20010)]. The cells were further disrupted by sonication for a total sonication “on” time of 5 min (10 × 10 s on/off) at 40% amplitude at 4°C. The whole cell lysate was cleared by centrifugation in a Ti45 rotor at 88,000*g* for 30 min at 4°C. A 5-ml slurry of anti-FLAG M2 affinity gel (Merck, A2220) was prepared by first washing with 50 ml of water and then 50 ml of buffer A [50 mM Hepes (pH 7.5 at 23°C), 150 mM NaCl, 100 mM l-arginine, and 10% (v/v) glycerol]. The following steps were all carried out in a cold room (4°C). The cleared lysate and the resin were incubated in a 250-ml centrifuge tube for 1 hour on a roller and then transferred to a 2.5 cm–by–10 cm glass Econo-Column (Bio-Rad, 7372512). The resin was then washed with 2 × 50 ml of buffer A followed by 2 × 50 ml of buffer B [50 mM Hepes (pH 7.5 at 23°C), 150 mM NaCl, and 10% (v/v) glycerol]. Five elution fractions were collected by gently resuspending the resin in 5 ml of elution buffer [buffer B supplemented with 3× FLAG peptide (200 μg/ml; Sigma-Aldrich, F4799)] and incubating for 10 min at 4°C. Additional buffer B was used for two further 5-ml elution fractions. The combined elution fractions were concentrated in a 100-kDa molecular weight cutoff (MWCO) centrifugal filter and lastly purified by size exclusion chromatography (Superose 6 16/600 column, GE HealthCare) using gel filtration buffer [50 mM Hepes (pH 7.5 at 23°C), 150 mM NaCl, 10% glycerol, and 2 mM (tris(2-carboxyethyl)phosphine) (TCEP)]. Purified protein was concentrated, aliquoted, flash-frozen in liquid nitrogen, and stored at −80°C.

### Expression and purification of His-MBP-TEV-p53 (1-102)

The p53 (residues 1 to 102) gene fragment was PCR-amplified from a plasmid containing human p53 (a gift from the Alan Fersht Laboratory) (*46*, *47*). Initially, the p53 fragment was cloned with an N-terminal glutathione *S*-transferase (GST) tag; however, the GST-p53 construct dimerizes via GST when used at high concentrations, affecting the kinase assay measurements. Therefore, the p53 fragment was cloned into an Nde I/Bam HI–cut pOPTHM(TEV) vector. The construct has an N-terminal 6× His tag followed by maltose-binding protein (MBP) and a TEV cleavage site.

A single colony from C41(DE3) RIPL cells transformed with the His-MBP-TEV-p53(1-102) plasmid was transferred to 150 ml of 2xTY with 150 μl of ampicillin (100 mg/ml). The culture was grown overnight at 37°C with shaking at 200 rpm then, the following morning, split into 12 × 900 ml of 2xTY to scale up growth (12 ml of overnight culture pipetted into each). Cells were grown to an optical density at 600 nm (OD_600_) of 0.8 (37°C and 200 rpm), induced with 0.5 mM isopropyl-β-d-thiogalactopyranoside (IPTG) per flask, and then left to shake for 3 hours (37°C and 200 rpm). Cells were harvested in 2-liter bottles lined with bags at 6000*g* at 4°C for 25 min. Excess medium was removed, and bags with pellets were flash-frozen in liquid nitrogen and stored at −80°C.

A cell pellet from 1.8 liters of cell culture was resuspended in lysis buffer [50 mM Hepes (pH 7.5), 150 mM NaCl, 10 mM imidazole, and 10% glycerol, supplemented with EDTA-free protease cocktail inhibitors (Roche 05056489001), Pierce Universal Nuclease (Thermo Fisher Scientific, 88702), and Pefabloc (Melford, A20010)]. Resuspended cells were disrupted by sonication for a total sonication on time of 5 min (10 × 10 s on/off) at 60% amplitude at 4°C. Cell lysate was cleared by spinning at 88,000*g* for 30 min at 4°C in a Ti45 rotor. The clarified supernatant was filtered using a 5-μm filter, then injected onto a 5-ml HisTrap column, and washed with buffer A [50 mM Hepes (pH 7.5 at 23°C), 150 mM NaCl, and 10 mM imidazole] before applying a 0 to 100% gradient elution from buffer A to buffer B [50 mM Hepes (pH 7.5 at 23°C), 150 mM NaCl, and 1 M imidazole]. His-MBP-TEV-p53(1-102) was eluted at around 120 mM imidazole. The peak fractions were pooled and diluted to a final NaCl concentration of 50 mM using Q dilution buffer [20 mM tris (pH 8.0 at 4°C)] before injection onto a 5-ml HiTrap Q Column equilibrated with buffer containing 20 mM tris (pH 8.0 at 4°C) and 50 mM NaCl. Protein was eluted with a 50 to 1000 mM NaCl gradient (p53 eluted at 180 mM NaCl). Fractions containing His-MBP-TEV-p53(1-102) were pooled, concentrated using a 30-kDa MWCO centrifugal filter unit, and then buffer-exchanged into 50 mM Hepes (pH 7.5 at 23°C), 150 mM NaCl, 10% glycerol, and 0.5 mM TCEP buffer using a PD10 column (GE HealthCare, 17-0851-01). The buffer-exchanged protein was concentrated, aliquoted, flash-frozen in liquid nitrogen, and stored at −80°C.

### Expression and purification of His-MBP-TEV-CHK2(1-107)

The gene fragment for CHK2 (residues 1 to 107) was codon-optimized for *Escherichia coli* expression and ordered from IDTDNA. The gene fragment was assembled into a pOPTHM(TEV) vector with an N-terminal 6× His tag followed by MBP and a TEV cleavage site.

A single colony from C41(DE3) RIPL cells transformed with the His-MBP-TEV-CHK2(1-107) plasmid was transferred to 100 ml of 2xTY with 100 μl of ampicillin (100 mg/ml). The culture was grown overnight at 37°C with shaking at 200 rpm then, the following morning, split into 2 × 900 ml of 2xTY to scale up growth (12 ml of overnight culture pipetted into each). Cells were grown to OD_600_ of 0.6 (37°C and 200 rpm), induced with 0.5 mM IPTG per flask, and then left to shake for 5 hours (37°C and 200 rpm). Cells were harvested in 2-liter bottles lined with bags at 6000*g* at 4°C for 25 min. Excess medium was removed, and bags with pellets were flash-frozen in liquid nitrogen and stored at −80°C.

A cell pellet from 1.8 liters of cell culture was resuspended in lysis buffer [25 mM tris (pH 8.0), 300 mM NaCl, 10 mM imidazole, and 5% glycerol, supplemented with EDTA-free protease cocktail inhibitors (Roche, 05056489001), Pierce Universal Nuclease (Thermo Fisher Scientific, 88702), and Pefabloc (Melford, A20010)]. Resuspended cells were disrupted by sonication for a total sonication on time of 5 min (5 × 5 s on/off) at 60% amplitude at 4°C. Cell lysate was cleared by spinning at 88,000*g* for 30 min at 4°C in a Ti45 rotor. The clarified supernatant was filtered using a 5-μm filter and then injected onto 2 × 5–ml HisTrap columns and washed with buffer A [50 mM Hepes (pH 7.5 at 23°C), 150 mM NaCl, and 10 mM imidazole] before applying a 0 to 100% gradient elution from buffer A to buffer B [50 mM Hepes (pH 7.5 at 23°C), 150 mM NaCl, and 1 M imidazole]. His-MBP-TEV-CHK2(1-107) was eluted at around 110 mM imidazole. The peak fractions were pooled and diluted to a final NaCl concentration of 50 mM using Q dilution buffer [20 mM tris (pH 8.0 at 4°C)] before injection onto a 5-ml HiTrap Q column equilibrated with buffer containing 20 mM tris (pH 8.0 at 4°C), 50 mM NaCl, and 10% glycerol. Protein was eluted with a 50 to 1000 mM NaCl gradient [His-MBP-TEV-CHK2(1-107) eluted at 190 mM]. Fractions containing His-MBP-TEV-CHK2(1-107) were pooled, concentrated using a 30-kDa MWCO centrifugal filter unit to 2 ml, and then injected onto a Superdex 200 HiLoad 16/60 prep grade column equilibrated with buffer containing 50 mM Hepes (pH 7.5 at 23°C), 150 mM NaCl, and 10% glycerol. His-MBP-TEV-CHK2(1-107) fractions were concentrated, aliquoted, flash-frozen in liquid nitrogen, and stored at −80°C.

### Expression and purification of His-MBP-TEV-NRF1

The gene fragment for full-length NRF1 was codon-optimized for *E. coli* expression and ordered from IDTDNA. The gene fragment was assembled into a pOPTHM(TEV) vector with an N-terminal 6× His tag followed by MBP and a TEV cleavage site.

A single colony from C41(DE3) RIPL cells transformed with the His-MBP-TEV-NRF1 plasmid was transferred to 100 ml of 2xTY with 100 μl of ampicillin (100 mg/ml). The culture was grown overnight at 37°C with shaking at 200 rpm then, the following morning, split into 2 × 900 ml of 2xTY to scale up growth (12 ml of overnight culture pipetted into each). Cells were grown to OD_600_ of 0.6 (37°C and 200 rpm), induced with 0.5 mM IPTG per flask, and then left to shake for 5 hours (37°C and 200 rpm). Cells were harvested in 2-liter bottles lined with bags at 6000*g* at 4°C for 25 min. Excess medium was removed, and bags with pellets were flash-frozen in liquid nitrogen and stored at −80°C.

A cell pellet from 1.8 liter of cell culture was resuspended in lysis buffer [25 mM tris (pH 8.0), 300 mM NaCl, 10 mM imidazole, and 5% glycerol, supplemented with EDTA-free protease cocktail inhibitors (Roche, 05056489001), Pierce Universal Nuclease (Thermo Fisher Scientific, 88702), and Pefabloc (Melford, A20010)]. Resuspended cells were disrupted by sonication for a total sonication on time of 4 min (2 × 2 s on/off) at 60% amplitude at 4°C. Cell lysate was cleared by spinning at 88,000*g* for 30 min at 4°C in a Ti45 rotor. The clarified supernatant was filtered using a 5-μm filter and then injected onto a 5-ml HisTrap column and washed with buffer A [25 mM tris (pH 8.0), 300 mM NaCl, 10 mM imidazole, and 5% glycerol] before applying a 0 to 100% gradient elution from buffer A to buffer B [25 mM tris (pH 7.5 at 23°C), 300 mM NaCl, 1 M imidazole, and 5% glycerol]. His-MBP-TEV-NRF1 was eluted at around 180 mM imidazole. The peak fractions were pooled and diluted to a final NaCl concentration of 100 mM using Hep dilution buffer [25 mM tris (pH 8.0 at 4°C) and 5% glycerol] before injection onto a 5-ml heparin column equilibrated with buffer containing 25 mM tris (pH 8.0 at 4°C), 100 mM NaCl, and 5% glycerol. Protein was eluted with a 50 to 1000 mM NaCl gradient (His-MBP-TEV-NRF1 eluted at 595 mM). Fractions containing His-MBP-TEV-NRF1 were pooled, concentrated using a 50-kDa MWCO centrifugal filter unit to 500 μl, and then injected onto a Superdex 200 30/100 column equilibrated with buffer containing 50 mM Hepes (pH 7.5 at 23°C), 150 mM NaCl, and 10% glycerol. His-MBP-TEV-NRF1 fractions were concentrated, aliquoted, flash-frozen in liquid nitrogen, and stored at −80°C.

### Expression and purification of recombinant human MRN

Codon-optimized sequences for full-length human Mre11, Rad50, and Nbs1 were cloned into vectors for insect cell expression using HiFi DNA assembly. Rad50, with a TEV-cleavable C-terminal twin Strep-tag II was cloned into pFastBac, while Mre11 (with no tags) and Nbs1 (with a C-terminal FRB domain followed by a TEV-cleavable twin Strep-tag II) were cloned in pFastBacDual, with Mre11 under PH promoter, and Nbs1 under P10 promoter. The bacmids and baculoviruses were generated using standard protocols (*48*). Transfections were carried out using Sf9 insect cells grown in antibiotic-free Sf-900 II SFM medium. Roughly 72 hours after transfection, the entire transfection culture (cells and medium) was harvested for the P1 virus. To make the P2 virus, Sf9 cells at 1 × 10^6^ cells/ml were infected with 4% (v/v) of P1 virus for 72 hours, after which the P2 virus was harvested by pelleting cell debris for 8 min at 4000*g* at 4°C. To generate the P3 virus, 100 ml of Sf9 cells at 2 × 10^6^ cells/ml were infected with 2% (v/v) of P2 virus, and after 72 hours, the virus was collected by centrifugation. For the MRN complex protein production, 2 liter of High Five cells at 1.5 × 10^6^ to 2.0 × 10^6^/ml density were infected with 10 ml of each P3 virus per 400 ml of High Five cells. Cells were harvested 48 hours after transfection by centrifugation, and pellets were flash-frozen in liquid nitrogen and stored at −80°C.

A cell pellet from 2 liter of culture was resuspended in 75 ml of cold lysis buffer [25 mM tris (pH 8.5 at 4°C), 300 mM NaCl, 100 mM arginine mono-HCl, and 10% glycerol (v/v), supplemented with EDTA-free protease cocktail inhibitor tablets (Roche, 05056489001), Universal Nuclease (Thermo Fisher Scientific, 88702), and Pefabloc (Melford, A20010)]. The cells were further disrupted by sonication for a total sonication on time of 2 min and 15 s (3-s on/8-s off) at 60% amplitude at 4°C. Cell lysate was cleared by centrifugation in a Ti45 rotor at 88,000*g* for 30 min at 4°C; then, clarified supernatant was filtered using 5-μm filters. Filtered supernatant was loaded onto 3 × 5–ml StrepTrap columns equilibrated with StrepTrap wash buffer [25 mM tris (pH 8.5 at 4°C), 300 mM NaCl, 100 mM arginine mono-HCl, and 10% glycerol (v/v)]. The columns were then washed with 4 × 10 ml of StrepTrap wash buffer. MRN was eluted using 6 × 15 ml of StrepTrap elution buffer [25 mM tris (pH 8.0 at 4°C), 150 mM NaCl, 10% glycerol (v/v), 10 mM desthiobiotin, and 0.5 M TCEP]. Elution fractions were pooled, and Q dilution buffer [25 mM tris (pH 7.5 at 4°C), 10% glycerol (v/v), and 0.5 M TCEP] was used to reduce the total NaCl concentration to 100 mM. Diluted and pooled elution fractions were loaded onto a 5-ml HiTrap Q column equilibrated with Q buffer A [25 mM tris (pH 7.5 at 4°C), 100 mM NaCl, 10% glycerol (v/v), and 0.5 M TCEP]. Step gradients of 2, 50, and 100% of Q buffer B [25 mM tris (pH 7.5 at 4°C), 1 M NaCl, 10% glycerol (v/v), and 0.5 M TCEP] were applied to the HiTrap Q column, with MRN eluting at 50% Q buffer B. MRN-containing fractions were pooled, concentrated to 2 ml using a 100-kDa MWCO centrifugal filter unit, and lastly purified by size exclusion chromatography (Superose 6 16/600 column, GE HealthCare) using gel filtration buffer [50 mM Hepes (pH 7.5 at 23°C), 300 mM NaCl, 10% glycerol, and 0.5 mM TCEP]. Purified protein was concentrated to 10 to 20 μM, aliquoted, flash-frozen with liquid nitrogen, and stored at −80°C.

### Substrate peptide

The 12-residue p53 substrate peptide (human p53 peptide 11-EPPL**S**QETFSDL-22) was custom-synthesized by Bankpeptide, China, with an N-terminal NH_2_ and C-terminal COOH, in acetate salt form, with trifluoroacetic acid removed. The lyophilized powder was dissolved to 2 mM in buffer containing 50 mM Hepes (pH 7.5), 100 mM NaCl, and 10% glycerol, aliquoted, flash-frozen, and stored at −80°C.

### Phos-tag gel assays

Phos-tag gels were cast in-house. The protocol followed was from the Wako Phos-tag SDS-PAGE Guidebook. Briefly, the resolving gel was made by gently mixing 7.5% acrylamide/bis-acrylamide (29:1, 3.3% cross-linker), 350 mM bis-tris/HCl (pH 6.8), 50 μM Phos-tag reagent (APExBIO, F4002), 100 μM ZnCl_2_, 0.1% tetramethylethylenediamine (TEMED) (v/v), and 0.05% ammonium persulfate (APS; w/v) in a 50-ml falcon tube. The solution was then pipetted between two glass plates (100 mm by 100 mm) that were separated by 1-mm spacers and sealed together on three sides by waterproof tape. The resolving gel was left to set for 20 min with a 70% ethanol overlay. The ethanol overlay was removed; then, the stacking gel solution was prepared by mixing 4.5% acrylamide/bis-acrylamide (29:1, 3.3% cross-linker), 350 mM bis-tris/HCl (pH 6.8), 0.1% TEMED (v/v), and 0.05% APS (w/v) in a 50-ml falcon tube. Stacking gel solution was then pipetted on top of the resolving gel in the cassette. Gel combs were placed into the stacking gel solution, and the gel was left to set for 20 min. Gels were run with 1× Mops buffer without EDTA (50 mM Mops, 50 mM tris base, and 3.5 mM SDS) for 1.5 to 2 hours at 150-V constant voltage.

All assays were run with 1 mM ATP and 5 mM MgCl_2_ at 30°C on a PCR block thermocycler or at room temperature (RT) on a PCR tube rack. Concentrations of components used in the assay are noted in the figure captions.

### ADP-Glo assay

The activity of ATM was measured using the ADP-Glo kinase assay kit (V9101, Promega). ATM wild type, ATM Δkα9b, and H_2_O_2_ were diluted to appropriate concentrations using kinase assay buffer [50 mM Hepes (pH 7.5 at 23°C), 100 mM NaCl, and 10 mM MgCl_2_]. Samples were assembled in 0.2-ml PCR tubes. The kinase reaction was initiated by adding ATP (a volume equal to the sample volume) to the sample tubes, and the reactions were carried out at 30°C on a PCR block thermocycler. Final concentrations of components after the addition of ATP were 250 nM ATM (for wild-type ATM) or 25 nM (for ATM Δkα9b), 0.5 mM H_2_O_2_, and 250 μM ATP. At 60 min, 5 μl of the samples were added to 5 μl of ADP-Glo reagent in a 384-well plate to stop the reaction and deplete the remaining ATP. After incubating for 40 min at RT, 10 μl of kinase detection reagent was added to the wells and incubated for a further 40 min at RT. The luminescence from each well was measured using a PHERAstar FSX microplate reader. Analysis and calculations were run in Prism 10. Luminescence measured for a buffer-only control was subtracted as background signal. An ATP-to-ADP standard conversion curve was used to convert luminescence signal to concentration of ADP generated; then, specific rates were calculated by adjusting for the ATM concentration. Three biological replicates were used.

### Determination of kinetic parameters

ATM kinase activity was measured by monitoring phosphorylation of the MBP-p53^102^ peptide substrate. Gels were stained using InstantBlue stain, and after destaining in water, gels were visualized using a ChemiDoc MP imaging system. Phosphorylated and nonphosphorylated substrate band intensities from Phos-tag gels were quantified using Bio-Rad Laboratories Image Lab version 6.0.1. Initial rates were calculated by measuring the ratio of phosphorylated substrate to nonphosphorylated substrate and then calculating the amount of substrate phosphorylation at four different time points in the linear range and adjusting for the enzyme concentration. Kinetic parameters *k*_cat_ and *K*_M_ were calculated using Prism 9 assuming Michaelis-Menten kinetics.

### Cryo-EM sample preparation

Samples (20 μl) were prepared in PCR tubes with 2 μM ATM Q2971A (with N-terminal FLAG-FKBP tag), 0.5 mM p53 peptide (residues 11 to 22), 1 mM H_2_O_2_, 1 mM AMP-PNP, and 5 mM MgCl_2_ using a dilution buffer of 50 mM Hepes (pH 7.5), 100 mM NaCl, and 10% glycerol. Samples were first incubated with all components except H_2_O_2_. Tubes were kept on ice for around 30 min while setting up an FEI Vitrobot Mk IV and glow discharging grids. The Vitrobot chamber was set to 30°C and 100% humidity. UltrAuFoil R1.2/1.3 Au 300 mesh grids were glow-discharged using a sputter coater discharger (Edwards, S150B) for 1 min and 15 s at 0.8 kV, 30 mA, and 10^−1^ torr. Sample tubes were moved to an RT holder and incubated at RT for 1 min; then, 2 μl of a 10 mM H_2_O_2_ stock was added to the tubes. Sample tubes were further incubated at RT between 7.5 and 15 min, and 4-μl droplets were pipetted onto grids. Grids were blotted using Whatman grade 1 filter paper with 0-s incubation time, 2-s blot time, and −7 blot force and vitrified in liquid ethane using an FEI Vitrobot Mk IV.

### Cryo-EM data collection

Three separate datasets were collected on a Thermo Fisher Scientific Titan Krios transmission electron microscope operated at 300 kV. This was equipped with a Gatan K3 direct electron detector and GIF quantum energy filter. EPU software was used for automatic data collection in counting superresolution mode with binning 2. The physical pixel size was 0.826 Å, and the total exposure time was 1.5 to 2.0 s, with a total dose of 34 to 45 e^−^/Å^2^, where doses were fractionated into 40 frames. A slit width of 20 eV was applied on the energy filter, and a defocus range of −1 to −3 μm was used for all sessions. A total of 53,113 movies were collected.

### Cryo-EM data processing

For each of the three datasets, initial processing of movies and particle extraction were carried out in the same way using RELION 3.1 (fig. S4) (*49*). Movies were imported, and movie frames were aligned using MOTIONCOR2 (*50*); then, contrast transfer function (CTF) estimation was performed using CTFFIND4 (*51*). Particles were picked using SPHIRE-crYOLO (*52*), and these coordinates were used for particle extraction in RELION. A total of 1,221,894 particles were extracted from dataset one; a total of 1,012,497 particles were extracted from dataset two, and 2,295,545 particles were extracted from dataset three. Particles stacks from each dataset were imported into cryoSPARC v3 (*53*), and heterogeneous refinement was performed using a single true map representing the ATM dimer and four or five noise decoy maps to select for ATM particles. To ensure that no potential ATM monomers were being ignored during these early stages of processing, two strategies were used to search for monomers. First, particles that were sorted into noise decoy classes were further subjected to 2D classification using 50 classes. The classes generated did not appear to have monomeric ATM or any other proteins with distinct features other than chaperone protein. Second, a monomer ATM map was generated by erasing one side of the ATM dimer in UCSF Chimera (*54*). This map and an ATM dimer map were then used in heterogeneous refinement in cryoSPARC v3 using all particles to search for ATM monomers; however, no distinct monomers could be found.

Particles from datasets one and two that were heterogeneously refined using the true ATM map were combined (dataset three had not been collected at this time), and the resulting 1,048,650 particles were used in nonuniform refinement. The particles from the nonuniform refinement were subsequently converted to STAR format using pyem (*55*) so that particles could be used in RELION. The particles were subjected to 3D classification, separating particles into six classes and limiting the resolution E-step to 7 Å, and for the reference map, the nonuniformly refined ATM map generated from combined particles was used. Particles were selected from three classes that gave the highest resolutions and where helices were well defined. These were used in a second round of 3D classification, splitting particles into six classes and limiting the resolution E-step to 7 Å, using the nonuniformly refined map and masking the C-terminal region from the dimer containing the FAT, pincer, and kinase domains. The best-resolved class map (class 6) appeared to have defined helices and could accommodate, with nearly no changes, a previously published apo-ATM model [Protein Data Bank (PDB): 7SIC]. The second best-resolved class map (class 5) was also broadly similar to the class 6 map. However, when both maps were viewed at the same threshold, the class 5 map had incomplete density for the FLAP-BE helices and the LBE (kα4b and kα4c) helices, and the helices for one protomer were slightly shifted compared to the other (the helices of PDB: 7SIC did not perfectly fit), which indicated that there might be additional flexibility to resolve in these regions.

Class 5 particles (95,314) were imported again into cryoSPARC and subjected to 3D variability analysis (*56*) to see whether different conformational states of ATM might be revealed. The job was run with three different modes to solve, and the results were filtered to a resolution of 6 Å, which should have been sufficient to differentiate movement at the level of helices. Three different volume series (series 0, 1, and 2) were output from a subsequent 3D variability display job and analyzed in Chimera. Series 2 demonstrated the most notable changes: Volume maps appeared to transition between the expected basal state of ATM and a more twisted state of the ATM dimer where one protomer of ATM appeared rotated with respect to the other. The twisted state and basal state maps that represented the extreme ends of volume series 2 were used as the two references in a heterogeneous refinement job, where all the class 5 particles were input to separate them into these two populations. The 34,796 particles that were sorted into the twisted state were used in a further nonuniform refinement job alongside the series 2 twisted state map and were resolved to a final resolution of 3.70 Å. The 60,518 particles that were sorted into the ATM basal state were nonuniformly refined to 3.25 Å.

Once dataset three had been collected, ATM particles from datasets one and two were combined with dataset three’s ATM particles (sorted by heterogeneous refinement) for a total of 2,256,344 particles that were nonuniformly refined to 3.07 Å. These were subsequently used in a heterogeneous refinement job that used two true maps (the volume series 2 nonuniformly refined maps of twisted and basal state ATM) and a noise decoy volume. A total of 1,207,435 particles were sorted into the basal state class; a total of 842,459 particles were sorted into the twisted state class, and the remaining particles were sorted into the noise class. The 1,207,435 particles sorted into the basal ATM class were nonuniformly refined against the previously generated 3.25-Å basal state map, resulting in a higher-resolution reconstruction of 2.79 Å. These particles were subsequently converted into STAR format for three rounds of CTF refinement and Bayesian polishing in RELION (*57*). Nonuniform refinement with C2 symmetry in cryoSPARC using the polished particles resulted in a resolution of 2.53 Å. Subtracting the N termini from these particles and running local refinement in cryoSPARC on the dimeric C-terminal region gave a final map with a resolution of 2.50 Å. The particles and 2.53-Å resolution map from the nonuniform refinement job were subjected to homogeneous refinement to obtain the consensus map at 2.61-Å resolution.

The 842,459 particles that were sorted into twisted state ATM were used in two further rounds of heterogeneous refinement (using the volume series 2 nonuniformly refined twisted and basal state maps alongside a noise decoy volume as references), and 616,279 particles were sorted into the twisted state ATM after the third round. One more round of heterogeneous refinement was run using these particles, although this time, two twisted state maps, two basal state maps, and two noise decoy volumes were used as the reference volumes. The best twisted state class contained 255,231 particles that could be nonuniformly refined to 3.32 Å. These particles were converted to STAR format for use in RELION, where they were 3D-classified into six classes using a regularization T-parameter of 20. The best class containing 30,707 particles was 3D-refined to 3.8 Å and then subjected to three rounds of CTF refinement and Bayesian polishing. These polished particles were imported back into cryoSPARC where they were nonuniformly refined with C2 symmetry to a resolution of 3.13 Å. As with the basal state map, particle subtraction and then local refinement were run for the dimeric C-terminal region in cryoSPARC, which gave a final resolution of 3.03 Å. To obtain a map for the N-terminal region of the twisted state, particle symmetry expansion (using C2 symmetry) and then signal subtraction and local refinement were run in cryoSPARC on the nonuniformly refined particles, and this gave a final resolution of 3.84 Å. The consensus map was generated by homogeneous refinement, using the nonuniformly refined particles and map, and gave a final resolution of 3.26 Å. The twisted state map is the H_2_O_2_-activated state map based on observations made here.

For the N-terminal region of the ATM basal state, 1,207,435 particles from the final basal state ATM refinement were subjected to symmetry expansion (applying C2 symmetry) in cryoSPARC (fig. S12). Signal was subtracted from the symmetry-expanded particles using a mask covering the dimeric C-terminal region and one N-terminal region. The signal-subtracted and symmetry-expanded particles were then 3D-classified with no alignment in RELION using a mask to focus on the remaining N-terminal region, and the class with the most complete N terminus (where the most helices were visible) with 185,535 particles was selected. These particles underwent local refinement in cryoSPARC, and a final map was obtained at 3.58-Å resolution.

### Model building and refinement

#### ATM basal state dimeric C-terminal region

A cryo-EM structure was available for ATM (PDB: 7SIC) (*21*), which we docked into the corresponding map density using rigid-body fitting in Chimera (*54*). Residues from the N termini that did not fit into the map density were deleted. Manual correction was then run in COOT (*58*). Real-space refinement in PHENIX (*59*) was run using default parameters in addition to adjusting the weight to 0.1 and targeting rotamer outliers using a sigma of 1.0, and an additional ANP restraints file was used as input alongside the map and model.

#### ATM H_2_O_2_-activated state dimeric C-terminal region

An available cryo-EM structure of ATM (PDB: 7SIC) was split into two separate monomers and fit into each side of the dimer map independently by rigid-body fitting in Chimera. Residues from the N termini that did not fit into the map density were deleted. The cryo-EM structure of SMG1-8-9 kinase bound to UPF1 peptide (*34*) (PDB: 6Z3R) was superimposed on one ATM protomer, aligning on the kinase C-lobes, and the peptide docked well into the additional density around the active site. The p53 peptide was built into this density. A copy of this peptide was rigid-body-fit into the other ATM protomer. ATM and p53 substrate peptides were merged into one model, and manual corrections were made in COOT. Real-space refinement in PHENIX was run using default parameters in addition to adjusting the weight to 0.1 and targeting rotamer outliers using a sigma of 1.0, and an additional ANP restraints file was used as input alongside the map and model.

#### ATM basal state consensus model

The N terminus from one ATM protomer (PDB: 7SIC) was rigid-body-fit into the locally refined basal state N terminus map density. Residues were removed, which could already be attributed to the basal state dimeric C-terminal region or the N terminus from the other protomer. Real-space refinement in PHENIX was run using default parameters in addition to adjusting the weight to 0.1 and targeting rotamer outliers using a sigma of 1.0. Two copies of the refined N terminus were rigid-body-fit into both sides of the ATM basal state consensus map density and merged with the model for the ATM basal state dimeric C-terminal region. Manual adjustments were made in COOT. Real-space refinement in PHENIX was run using default parameters in addition to adjusting the weight to 0.1 and targeting rotamer outliers using a sigma of 1.0, and an additional ANP restraints file was used as input alongside the map and model.

#### ATM H_2_O_2_-activated state consensus model

The refined N terminus for the basal state was rigid-body-fit into the locally refined H_2_O_2_-activated state N terminus map density. Real-space refinement in PHENIX was run using default parameters in addition to adjusting the weight to 0.1 and targeting rotamer outliers using a sigma of 1.0. This refined N terminus was then rigid-body-fit into both protomers of the ATM H_2_O_2_-activated consensus map density and merged with the model for the ATM H_2_O_2_-activated state dimeric C-terminal region. Manual adjustments were made in COOT. Real-space refinement in PHENIX was run using default parameters in addition to adjusting the weight to 0.1 and targeting rotamer outliers using a sigma of 1.0, and an additional ANP restraints file was used as input alongside the map and model.

## Supplementary Materials

### This PDF file includes:

Figs. S1 to S14 Tables S1 to S3 Legends for movies S1 to S5 References

## References

[1] C. Rothblum-Oviatt, J. Wright, M. A. Lefton-Greif, S. A. McGrath-Morrow, T. O. Crawford, H. M. Lederman, Ataxia telangiectasia: A review. Orphanet J. Rare Dis. 11, 159 (2016). 27884168 10.1186/s13023-016-0543-7 PMC5123280

[2] N. Takao, Y. Li, K. Yamamoto, Protective roles for ATM in cellular response to oxidative stress. FEBS Lett. 472, 133–136 (2000). 10781820 10.1016/s0014-5793(00)01422-8

[3] J. Reichenbach, R. Schubert, D. Schindler, K. Muller, H. Bohles, S. Zielen, Elevated oxidative stress in patients with ataxia telangiectasia. Antioxid. Redox Signal. 4, 465–469 (2002). 12215213 10.1089/15230860260196254

[4] G. Rotman, Y. Shiloh, The ATM gene and protein: Possible roles in genome surveillance, checkpoint controls and cellular defence against oxidative stress. Cancer Surv. 29, 285–304 (1997). 9338105

[5] G. Rotman, Y. Shiloh, Hypothesis: Ataxia-telangiectasia: Is ATM a sensor of oxidative damage and stress? Bioessays 19, 911–917 (1997). 9363685 10.1002/bies.950191011

[6] M. Ambrose, J. V. Goldstine, R. A. Gatti, Intrinsic mitochondrial dysfunction in ATM-deficient lymphoblastoid cells. Hum. Mol. Genet. 16, 2154–2164 (2007). 17606465 10.1093/hmg/ddm166

[7] H. M. Chow, A. Cheng, X. Song, M. R. Swerdel, R. P. Hart, K. Herrup, ATM is activated by ATP depletion and modulates mitochondrial function through NRF1. J. Cell Biol. 218, 909–928 (2019). 30642892 10.1083/jcb.201806197 PMC6400560

[8] J. H. Lee, M. R. Mand, C. H. Kao, Y. Zhou, S. W. Ryu, A. L. Richards, J. J. Coon, T. T. Paull, ATM directs DNA damage responses and proteostasis via genetically separable pathways. Sci. Signal. 11, (2018). 10.1126/scisignal.aan5598 PMC5898228 29317520

[9] Y. A. Valentin-Vega, K. H. Maclean, J. Tait-Mulder, S. Milasta, M. Steeves, F. C. Dorsey, J. L. Cleveland, D. R. Green, M. B. Kastan, Mitochondrial dysfunction in ataxia-telangiectasia. Blood 119, 1490–1500 (2012). 22144182 10.1182/blood-2011-08-373639 PMC3286212

[10] Z. Guo, S. Kozlov, M. F. Lavin, M. D. Person, T. T. Paull, ATM activation by oxidative stress. Science 330, 517–521 (2010). 20966255 10.1126/science.1192912

[11] R. E. Shackelford, C. L. Innes, S. O. Sieber, A. N. Heinloth, S. A. Leadon, R. S. Paules, The Ataxia telangiectasia gene product is required for oxidative stress-induced G1 and G2 checkpoint function in human fibroblasts. J. Biol. Chem. 276, 21951–21959 (2001). 11290740 10.1074/jbc.M011303200

[12] E. F. Fang, H. Kassahun, D. L. Croteau, M. Scheibye-Knudsen, K. Marosi, H. Lu, R. A. Shamanna, S. Kalyanasundaram, R. C. Bollineni, M. A. Wilson, W. B. Iser, B. N. Wollman, M. Morevati, J. Li, J. S. Kerr, Q. Lu, T. B. Waltz, J. Tian, D. A. Sinclair, M. P. Mattson, H. Nilsen, V. A. Bohr, NAD(+) replenishment improves lifespan and healthspan in ataxia telangiectasia models via mitophagy and DNA repair. Cell Metab. 24, 566–581 (2016). 27732836 10.1016/j.cmet.2016.09.004 PMC5777858

[13] J. H. Lee, T. T. Paull, Mitochondria at the crossroads of ATM-mediated stress signaling and regulation of reactive oxygen species. Redox Biol. 32, 101511 (2020). 32244177 10.1016/j.redox.2020.101511 PMC7115119

[14] C. Cosentino, D. Grieco, V. Costanzo, ATM activates the pentose phosphate pathway promoting anti-oxidant defence and DNA repair. EMBO J. 30, 546–555 (2011). 21157431 10.1038/emboj.2010.330 PMC3034007

[15] C. R. Reczek, N. S. Chandel, The two faces of reactive oxygen species in cancer. Annu. Rev. Cancer Biol. 1, 79–98 (2017). 10.1146/annurev-cancerbio-041916-065808 PMC11875389 40034143

[16] D. Baretic, R. L. Williams, PIKKs—The solenoid nest where partners and kinases meet. Curr. Opin. Struct. Biol. 29, 134–142 (2014). 25460276 10.1016/j.sbi.2014.11.003

[17] J. Perry, N. Kleckner, The ATRs, ATMs, and TORs are giant HEAT repeat proteins. Cell 112, 151–155 (2003). 12553904 10.1016/s0092-8674(03)00033-3

[18] D. Baretic, H. K. Pollard, D. I. Fisher, C. M. Johnson, B. Santhanam, C. M. Truman, T. Kouba, A. R. Fersht, C. Phillips, R. L. Williams, Structures of closed and open conformations of dimeric human ATM. Sci. Adv. 3, e1700933 (2017). 28508083 10.1126/sciadv.1700933 PMC5425235

[19] W. C. Lau, Y. Li, Z. Liu, Y. Gao, Q. Zhang, M. S. Huen, Structure of the human dimeric ATM kinase. Cell Cycle 15, 1117–1124 (2016). 27097373 10.1080/15384101.2016.1158362 PMC4889239

[20] K. Stakyte, M. Rotheneder, K. Lammens, J. D. Bartho, U. Gradler, T. Fuchss, U. Pehl, A. Alt, E. van de Logt, K. P. Hopfner, Molecular basis of human ATM kinase inhibition. Nat. Struct. Mol. Biol. 28, 789–798 (2021). 34556870 10.1038/s41594-021-00654-x

[21] C. Warren, N. P. Pavletich, Structure of the human ATM kinase and mechanism of Nbs1 binding. eLife 11, e74218 (2022). 35076389 10.7554/eLife.74218 PMC8828054

[22] J. Xiao, M. Liu, Y. Qi, Y. Chaban, C. Gao, B. Pan, Y. Tian, Z. Yu, J. Li, P. Zhang, Y. Xu, Structural insights into the activation of ATM kinase. Cell Res. 29, 683–685 (2019). 31320732 10.1038/s41422-019-0205-0 PMC6796860

[23] X. Wang, H. Chu, M. Lv, Z. Zhang, S. Qiu, H. Liu, X. Shen, W. Wang, G. Cai, Structure of the intact ATM/Tel1 kinase. Nat. Commun. 7, 11655 (2016). 27229179 10.1038/ncomms11655 PMC4894967

[24] M. Jansma, C. Linke-Winnebeck, S. Eustermann, K. Lammens, D. Kostrewa, K. Stakyte, C. Litz, B. Kessler, K. P. Hopfner, Near-complete structure and model of Tel1ATM from Chaetomium thermophilum reveals a robust autoinhibited ATP state. Structure 28, 83–95.e5 (2020). 31740028 10.1016/j.str.2019.10.013

[25] M. Sawicka, P. H. Wanrooij, V. C. Darbari, E. Tannous, S. Hailemariam, D. Bose, A. V. Makarova, P. M. Burgers, X. Zhang, The dimeric architecture of checkpoint kinases Mec1ATR and Tel1ATM reveal a common structural organization. J. Biol. Chem. 291, 13436–13447 (2016). 27129217 10.1074/jbc.M115.708263 PMC4919432

[26] J. Xin, Z. Xu, X. Wang, Y. Tian, Z. Zhang, G. Cai, Structural basis of allosteric regulation of Tel1/ATM kinase. Cell Res. 29, 655–665 (2019). 31097817 10.1038/s41422-019-0176-1 PMC6796912

[27] L. A. Yates, R. M. Williams, S. Hailemariam, R. Ayala, P. Burgers, X. Zhang, Cryo-EM structure of nucleotide-bound Tel1(ATM) unravels the molecular basis of inhibition and structural rationale for disease-associated mutations. Structure 28, 96–104.e3 (2020). 31740029 10.1016/j.str.2019.10.012 PMC6945111

[28] H. Yang, X. Jiang, B. Li, H. J. Yang, M. Miller, A. Yang, A. Dhar, N. P. Pavletich, Mechanisms of mTORC1 activation by RHEB and inhibition by PRAS40. Nature 552, 368–373 (2017). 29236692 10.1038/nature25023 PMC5750076

[29] C. J. Bakkenist, M. B. Kastan, DNA damage activates ATM through intermolecular autophosphorylation and dimer dissociation. Nature 421, 499–506 (2003). 12556884 10.1038/nature01368

[30] J. H. Lee, T. T. Paull, Direct activation of the ATM protein kinase by the Mre11/Rad50/Nbs1 complex. Science 304, 93–96 (2004). 15064416 10.1126/science.1091496

[31] L. O'Donoghue, A. Smolenski, Analysis of protein phosphorylation using Phos-tag gels. J. Proteomics 259, 104558 (2022). 35283355 10.1016/j.jprot.2022.104558

[32] J. H. Lee, T. T. Paull, ATM activation by DNA double-strand breaks through the Mre11-Rad50-Nbs1 complex. Science 308, 551–554 (2005). 15790808 10.1126/science.1108297

[33] C. Cirotti, S. Rizza, P. Giglio, N. Poerio, M. F. Allega, G. Claps, C. Pecorari, J. H. Lee, B. Benassi, D. Barilà, C. Robert, J. S. Stamler, F. Cecconi, M. Fraziano, T. T. Paull, G. Filomeni, Redox activation of ATM enhances GSNOR translation to sustain mitophagy and tolerance to oxidative stress. EMBO Rep. 22, e50500 (2021). 33245190 10.15252/embr.202050500 PMC7788447

[34] L. M. Langer, Y. Gat, F. Bonneau, E. Conti, Structure of substrate-bound SMG1-8-9 kinase complex reveals molecular basis for phosphorylation specificity. eLife 9, e57127 (2020). 32469312 10.7554/eLife.57127 PMC7334022

[35] S. T. Kim, D. S. Lim, C. E. Canman, M. B. Kastan, Substrate specificities and identification of putative substrates of ATM kinase family members. J. Biol. Chem. 274, 37538–37543 (1999). 10608806 10.1074/jbc.274.53.37538

[36] J. L. Johnson, T. M. Yaron, E. M. Huntsman, A. Kerelsky, J. Song, A. Regev, T. Y. Lin, K. Liberatore, D. M. Cizin, B. M. Cohen, N. Vasan, Y. Ma, K. Krismer, J. T. Robles, B. van de Kooij, A. E. van Vlimmeren, N. Andrée-Busch, N. F. Käufer, M. V. Dorovkov, A. G. Ryazanov, Y. Takagi, E. R. Kastenhuber, M. D. Goncalves, B. D. Hopkins, O. Elemento, D. J. Taatjes, A. Maucuer, A. Yamashita, A. Degterev, M. Uduman, J. Lu, S. D. Landry, B. Zhang, I. Cossentino, R. Linding, J. Blenis, P. V. Hornbeck, B. E. Turk, M. B. Yaffe, L. C. Cantley, An atlas of substrate specificities for the human serine/threonine kinome. Nature 613, 759–766 (2023). 36631611 10.1038/s41586-022-05575-3 PMC9876800

[37] T. O'Neill, A. J. Dwyer, Y. Ziv, D. W. Chan, S. P. Lees-Miller, R. H. Abraham, J. H. Lai, D. Hill, Y. Shiloh, L. C. Cantley, G. A. Rathbun, Utilization of oriented peptide libraries to identify substrate motifs selected by ATM. J. Biol. Chem. 275, 22719–22727 (2000). 10801797 10.1074/jbc.M001002200

[38] E. A. Tannous, L. A. Yates, X. Zhang, P. M. Burgers, Mechanism of auto-inhibition and activation of Mec1(ATR) checkpoint kinase. Nat. Struct. Mol. Biol. 28, 50–61 (2021). 33169019 10.1038/s41594-020-00522-0 PMC7855233

[39] S. Liang, T. L. Blundell, Human DNA-dependent protein kinase activation mechanism. Nat. Struct. Mol. Biol. 30, 140–147 (2023). 36604499 10.1038/s41594-022-00881-w PMC9935390

[40] X. Chen, X. Xu, Y. Chen, J. C. Cheung, H. Wang, J. Jiang, N. de Val, T. Fox, M. Gellert, W. Yang, Structure of an activated DNA-PK and its implications for NHEJ. Mol. Cell 81, 801–810.e3 (2021). 33385326 10.1016/j.molcel.2020.12.015 PMC7897279

[41] A. Corcoran, T. G. Cotter, Redox regulation of protein kinases. FEBS J. 280, 1944–1965 (2013). 23461806 10.1111/febs.12224

[42] T. H. Truong, K. S. Carroll, Redox regulation of protein kinases. Crit. Rev. Biochem. Mol. Biol. 48, 332–356 (2013). 23639002 10.3109/10409238.2013.790873 PMC4358782

[43] S. V. Kozlov, A. J. Waardenberg, K. Engholm-Keller, J. W. Arthur, M. E. Graham, M. Lavin, Reactive oxygen species (ROS)-activated ATM-dependent phosphorylation of cytoplasmic substrates identified by large-scale phosphoproteomics screen. Mol. Cell. Proteomics 15, 1032–1047 (2016). 26699800 10.1074/mcp.M115.055723 PMC4813686

[44] J. H. Lee, T. T. Paull, Cellular functions of the protein kinase ATM and their relevance to human disease. Nat. Rev. Mol. Cell Biol. 22, 796–814 (2021). 34429537 10.1038/s41580-021-00394-2

[45] K. Li, G. Bronk, J. Kondev, J. E. Haber, Yeast ATM and ATR kinases use different mechanisms to spread histone H2A phosphorylation around a DNA double-strand break. Proc. Natl. Acad. Sci. U.S.A. 117, 21354–21363 (2020). 32817543 10.1073/pnas.2002126117 PMC7474660

[46] A. C. Joerger, M. D. Allen, A. R. Fersht, Crystal structure of a superstable mutant of human p53 core domain. Insights into the mechanism of rescuing oncogenic mutations. J. Biol. Chem. 279, 1291–1296 (2004). 14534297 10.1074/jbc.M309732200

[47] P. V. Nikolova, J. Henckel, D. P. Lane, A. R. Fersht, Semirational design of active tumor suppressor p53 DNA binding domain with enhanced stability. Proc. Natl. Acad. Sci. U.S.A. 95, 14675–14680 (1998). 9843948 10.1073/pnas.95.25.14675 PMC24508

[48] Z. Zhang, J. Yang, D. Barford, Recombinant expression and reconstitution of multiprotein complexes by the USER cloning method in the insect cell-baculovirus expression system. Methods 95, 13–25 (2016). 26454197 10.1016/j.ymeth.2015.10.003

[49] J. Zivanov, T. Nakane, B. O. Forsberg, D. Kimanius, W. J. Hagen, E. Lindahl, S. H. Scheres, New tools for automated high-resolution cryo-EM structure determination in RELION-3. eLife 7, e42166 (2018). 30412051 10.7554/eLife.42166 PMC6250425

[50] S. Q. Zheng, E. Palovcak, J. P. Armache, K. A. Verba, Y. Cheng, D. A. Agard, MotionCor2: Anisotropic correction of beam-induced motion for improved cryo-electron microscopy. Nat. Methods 14, 331–332 (2017). 28250466 10.1038/nmeth.4193 PMC5494038

[51] A. Rohou, N. Grigorieff, CTFFIND4: Fast and accurate defocus estimation from electron micrographs. J. Struct. Biol. 192, 216–221 (2015). 26278980 10.1016/j.jsb.2015.08.008 PMC6760662

[52] T. Wagner, F. Merino, M. Stabrin, T. Moriya, C. Antoni, A. Apelbaum, P. Hagel, O. Sitsel, T. Raisch, D. Prumbaum, D. Quentin, D. Roderer, S. Tacke, B. Siebolds, E. Schubert, T. R. Shaikh, P. Lill, C. Gatsogiannis, S. Raunser, SPHIRE-crYOLO is a fast and accurate fully automated particle picker for cryo-EM. Commun. Biol. 2, 218 (2019). 31240256 10.1038/s42003-019-0437-z PMC6584505

[53] A. Punjani, J. L. Rubinstein, D. J. Fleet, M. A. Brubaker, cryoSPARC: Algorithms for rapid unsupervised cryo-EM structure determination. Nat. Methods 14, 290–296 (2017). 28165473 10.1038/nmeth.4169

[54] E. F. Pettersen, T. D. Goddard, C. C. Huang, G. S. Couch, D. M. Greenblatt, E. C. Meng, T. E. Ferrin, UCSF Chimera—A visualization system for exploratory research and analysis. J. Comput. Chem. 25, 1605–1612 (2004). 15264254 10.1002/jcc.20084

[55] D. Asarnow, E. Palovcak, Y. Cheng, UCSF pyem v0.5. Zenodo. (2019).

[56] A. Punjani, D. J. Fleet, 3D variability analysis: Resolving continuous flexibility and discrete heterogeneity from single particle cryo-EM. J. Struct. Biol. 213, 107702 (2021). 33582281 10.1016/j.jsb.2021.107702

[57] J. Zivanov, T. Nakane, S. H. W. Scheres, A Bayesian approach to beam-induced motion correction in cryo-EM single-particle analysis. IUCrJ 6, 5–17 (2019). 10.1107/S205225251801463X PMC6327179 30713699

[58] P. Emsley, B. Lohkamp, W. G. Scott, K. Cowtan, Features and development of Coot. Acta Crystallogr. D Biol. Crystallogr. 66, 486–501 (2010). 20383002 10.1107/S0907444910007493 PMC2852313

[59] P. D. Adams, P. V. Afonine, G. Bunkoczi, V. B. Chen, I. W. Davis, N. Echols, J. J. Headd, L. W. Hung, G. J. Kapral, R. W. Grosse-Kunstleve, A. J. McCoy, N. W. Moriarty, R. Oeffner, R. J. Read, D. C. Richardson, J. S. Richardson, T. C. Terwilliger, P. H. Zwart, PHENIX: A comprehensive python-based system for macromolecular structure solution. Acta Crystallogr. D Biol. Crystallogr. 66, 213–221 (2010). 20124702 10.1107/S0907444909052925 PMC2815670

[60] I. Hickson, Y. Zhao, C. J. Richardson, S. J. Green, N. M. Martin, A. I. Orr, P. M. Reaper, S. P. Jackson, N. J. Curtin, G. C. Smith, Identification and characterization of a novel and specific inhibitor of the ataxia-telangiectasia mutated kinase ATM. Cancer Res. 64, 9152–9159 (2004). 15604286 10.1158/0008-5472.CAN-04-2727

[61] Y. Z. Tan, P. R. Baldwin, J. H. Davis, J. R. Williamson, C. S. Potter, B. Carragher, D. Lyumkis, Addressing preferred specimen orientation in single-particle cryo-EM through tilting. Nat. Methods 14, 793–796 (2017). 28671674 10.1038/nmeth.4347 PMC5533649

[62] J. Rozewicki, S. Li, K. M. Amada, D. M. Standley, K. Katoh, MAFFT-DASH: Integrated protein sequence and structural alignment. Nucleic Acids Res. 47, W5–w10 (2019). 31062021 10.1093/nar/gkz342 PMC6602451

[63] A. M. Waterhouse, J. B. Procter, D. M. Martin, M. Clamp, G. J. Barton, Jalview Version 2—a multiple sequence alignment editor and analysis workbench. Bioinformatics 25, 1189–1191 (2009). 19151095 10.1093/bioinformatics/btp033 PMC2672624

[64] X. Robert, P. Gouet, Deciphering key features in protein structures with the new ENDscript server. Nucleic Acids Res. 42, W320–W324 (2014). 24753421 10.1093/nar/gku316 PMC4086106

