# Supplementary material for: Structural insights into the activation of ataxia-telangiectasia mutated by oxidative stress
Source: Sci Adv. 2023 Sep 27;9(39):eadi8291. doi: 10.1126/sciadv.adi8291 (PMC10530080; doi:10.1126/sciadv.adi8291)
Supplement: Supplementary file 1 — Figs. S1 to S14 Tables S1 to S3 Legends for movies S1 to S5 References [file sciadv.adi8291_sm.pdf]

Science Advances
AAAS

Supplementary Materials for

Structural Insights into the Activation of Ataxia-Telangiectasia Mutated by Oxidative Stress

Anna C. Howes *et al.*

Corresponding author: Roger L. Williams, rlw@mrc-lmb.cam.ac.uk

Sci. Adv. **9**, eadi8291 (2023)
DOI: 10.1126/sciadv.adi8291

The PDF file includes:

Figs. S1 to S14
Tables S1 to S3
Legends for movies S1 to S5
References

Other Supplementary Material for this manuscript includes the following:

Movies S1 to S5

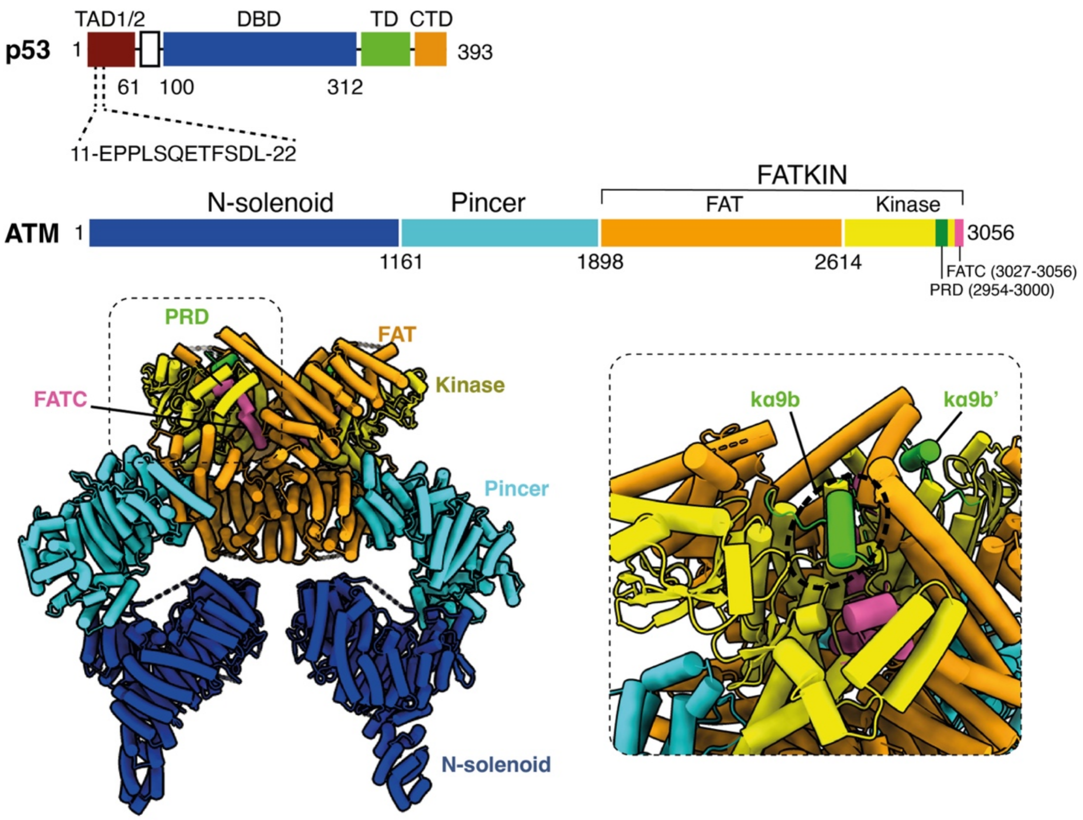

Fig. S1. Domain organization of p53 substrate and the structure of ATM kinase. The human p53 peptide sequence (residues 11-22, within the transactivation domain (TAD1) used in our cryo-EM sample is highlighted on a bar diagram showing the p53 transactivation domains (TAD1 and TAD2), DNA-binding domain (DBD), tetramerization domain (TD) and C-terminal domain (CTD), The domain architecture and structure of ATM (PDB: 7SIC) illustrate the locations of the N-solenoid, Pincer, FAT, kinase domain and FATC. Inset: Close-up image of the PRD kα9b helix.

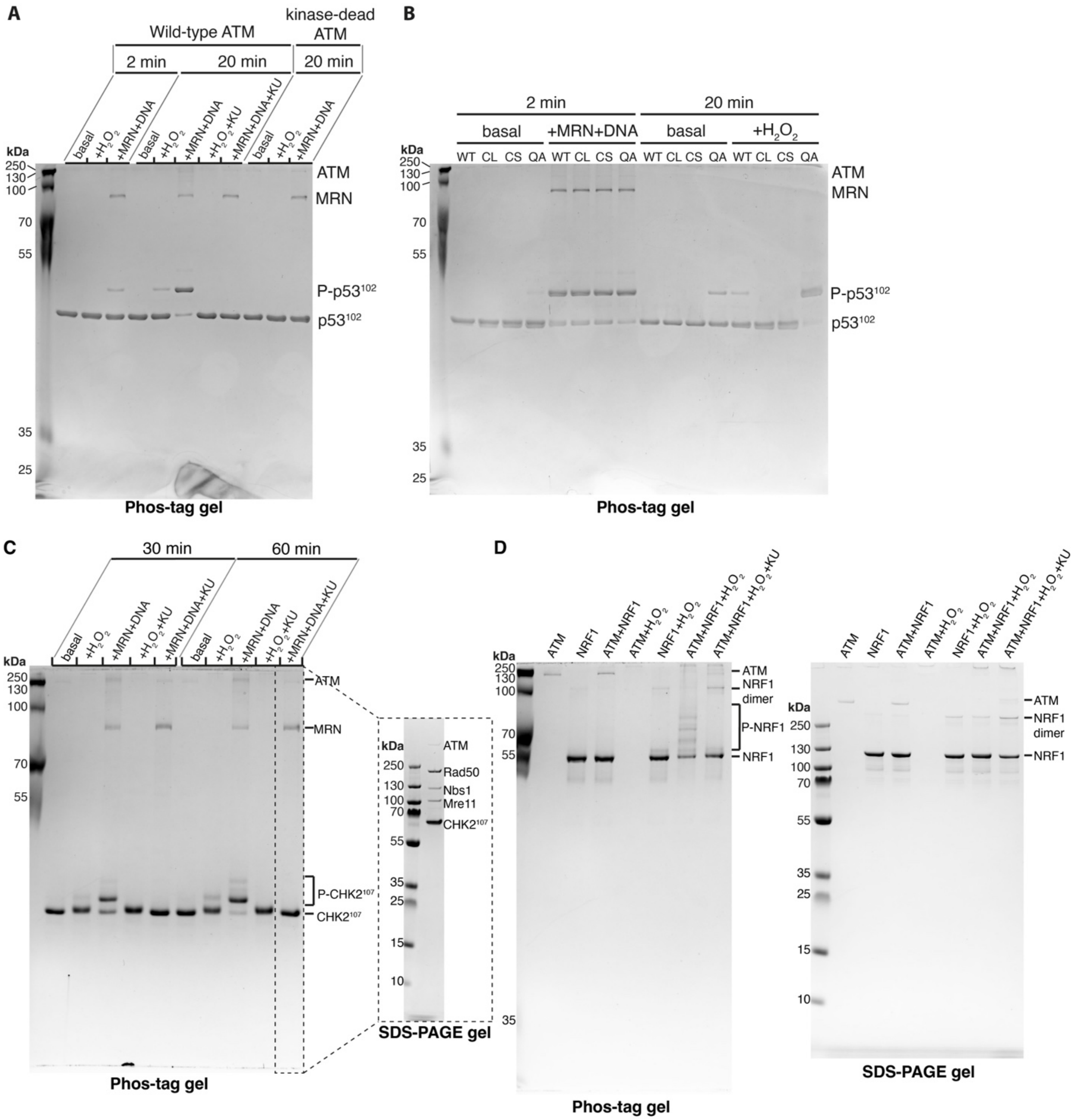

Fig. S2. ATM kinase activity measured by substrate phosphorylation on Phos-tag gels. ATM kinase activity was assessed by analyzing substrate phosphorylation on 7.5% acrylamide Phos-tag gels. Samples were incubated at 30 ˚C before being quenched with LDS sample buffer. Three biological replicates were run for these experiments. For (**A**) and (**B**), assays used 25 nM ATM (wild-type or mutant) incubated without activator (basal), with 0.5 mM H_2O_2, or with 250 nM MRN and 10 nM 350 bp dsDNA. All samples contained 5 µM MBP-p53(1-102) (p53^{102}), 1 mM ATP and 5 mM $MgCl_2$. (**A**) Monitoring p53^{102} phosphorylation for WT ATM – either basal, H_2O_2-activated or MRN/DNA activated. Use of 1 mM KU55933 inhibitor (KU for short) *(60)* or kinase-dead (D2870A/N2875K) ATM results in no substrate phosphorylation. (**B**) C2991L (CL), C2991S (CS) and Q2971A (QA) mutants were compared to wild-type (WT) ATM**.** (**C**) For

phosphorylation of CHK2(1-107), 25 nM ATM was incubated without activator (basal), with 0.5 mM H_2O_2, or with 250 nM MRN and 10 nM 350 bp dsDNA, and 1 mM KU55933 inhibitor when used. All samples contained 5 µM MBP-CHK2(1-107) ($CHK2^{107}$), 1 mM ATP and 5 mM $MgCl_2$. (**D**) Phosphorylation of full-length NRF1. Assays contained 100 nM ATM, 0.5 mM H_2O_2, 5 µM MBP-NRF1, 1 mM ATP and 5 mM $MgCl_2$. Samples were run either on a Phos-tag gel (left) or SDS-PAGE gel (right).

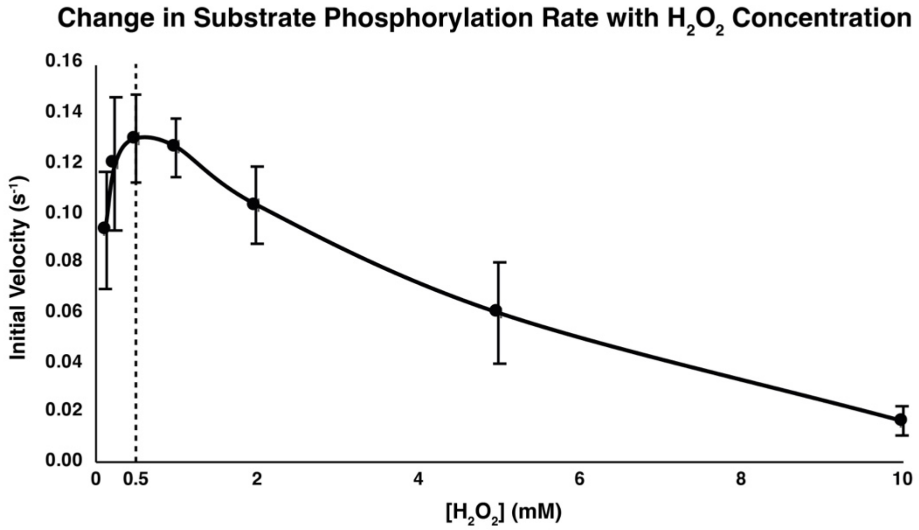

	Initial velocity (s^{-1})		
H_2O_2 concentration (mM)	1	2	3
0.125	0.08	0.12	0.08
0.25	0.10	0.15	0.11
0.5	0.12	0.15	0.12
1	0.12	0.14	0.12
2	0.10	0.12	0.09
5	0.06	0.08	0.04
10	0.02	0.02	0.01

Fig. S3. Initial velocities for MBP-p53(1-102) substrate phosphorylation by ATM plotted against increasing H_2O_2 concentration. 50 nM wild-type ATM was incubated with 25 µM MBP-p53(1-102), 1 mM ATP, 5 mM $MgCl_2$, and 0.125, 0.25, 0.5, 1, 2, 5, or 10 mM H_2O_2. Samples were incubated at 30 ˚C and the incubation time for each H_2O_2 concentration was adjusted to fall within the linear range of kinase activity. Reactions were stopped with LDS sample buffer. Data show mean (n = 3, biological replicates) ± standard deviation. Data values are displayed in a table below the graph.

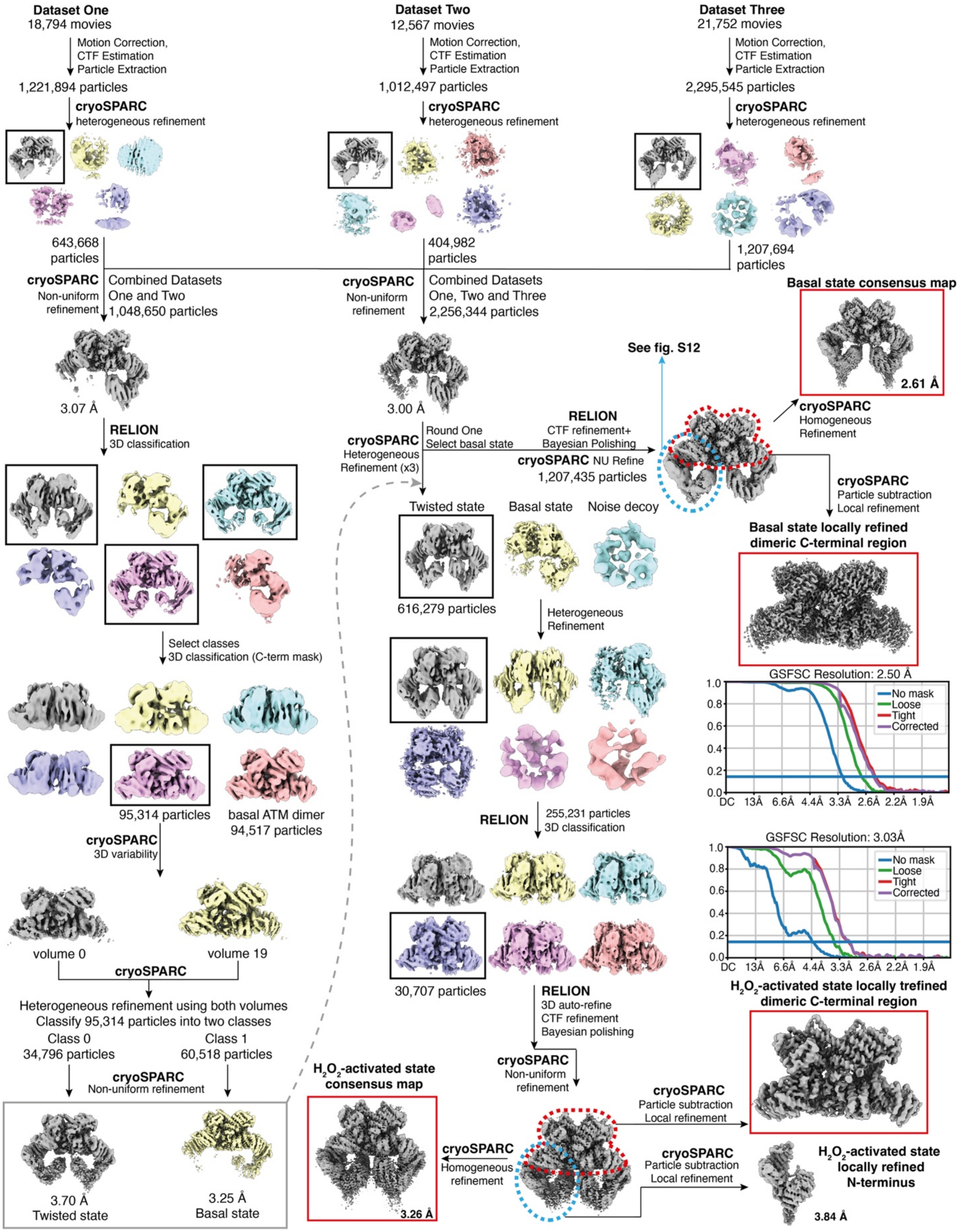

Fig. S4. Cryo-EM single particle analysis data processing workflow.

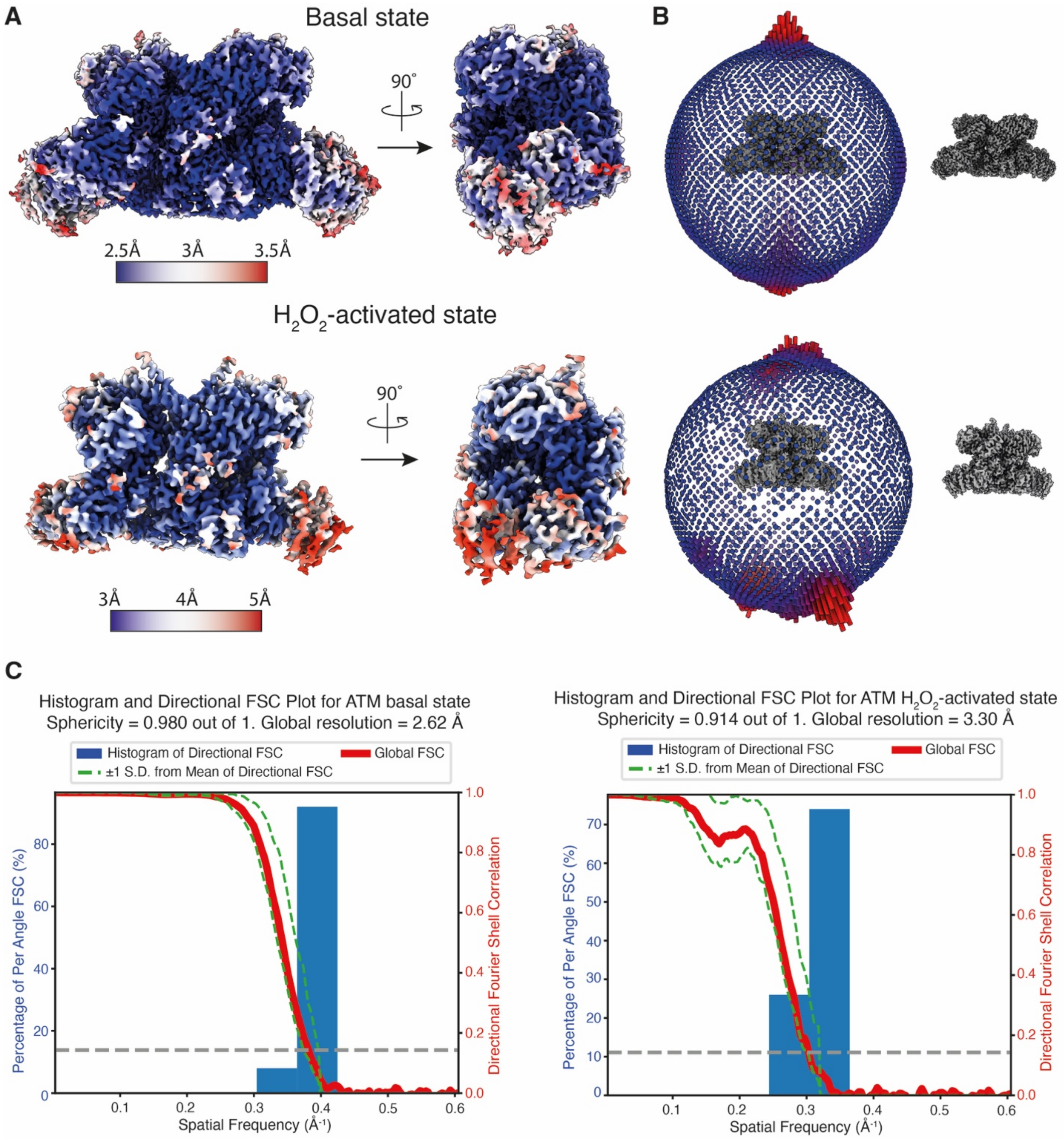

Fig. S5. Cryo-EM map quality metrics for the ATM basal state and H_2O_2-activated state dimer C-terminus maps. (**A**) Front and side views of sharpened EM density maps colored by local resolution, estimated using cryoSPARC v4. (**B**) Euler angle distribution for particles contributing to the final 3D reconstructions. EM density maps are shown separately to the right for clearer illustration of the map orientation. (**C**) Directional FSC plots and histograms for the final reconstructions calculated using 3DFSC *(61)*.

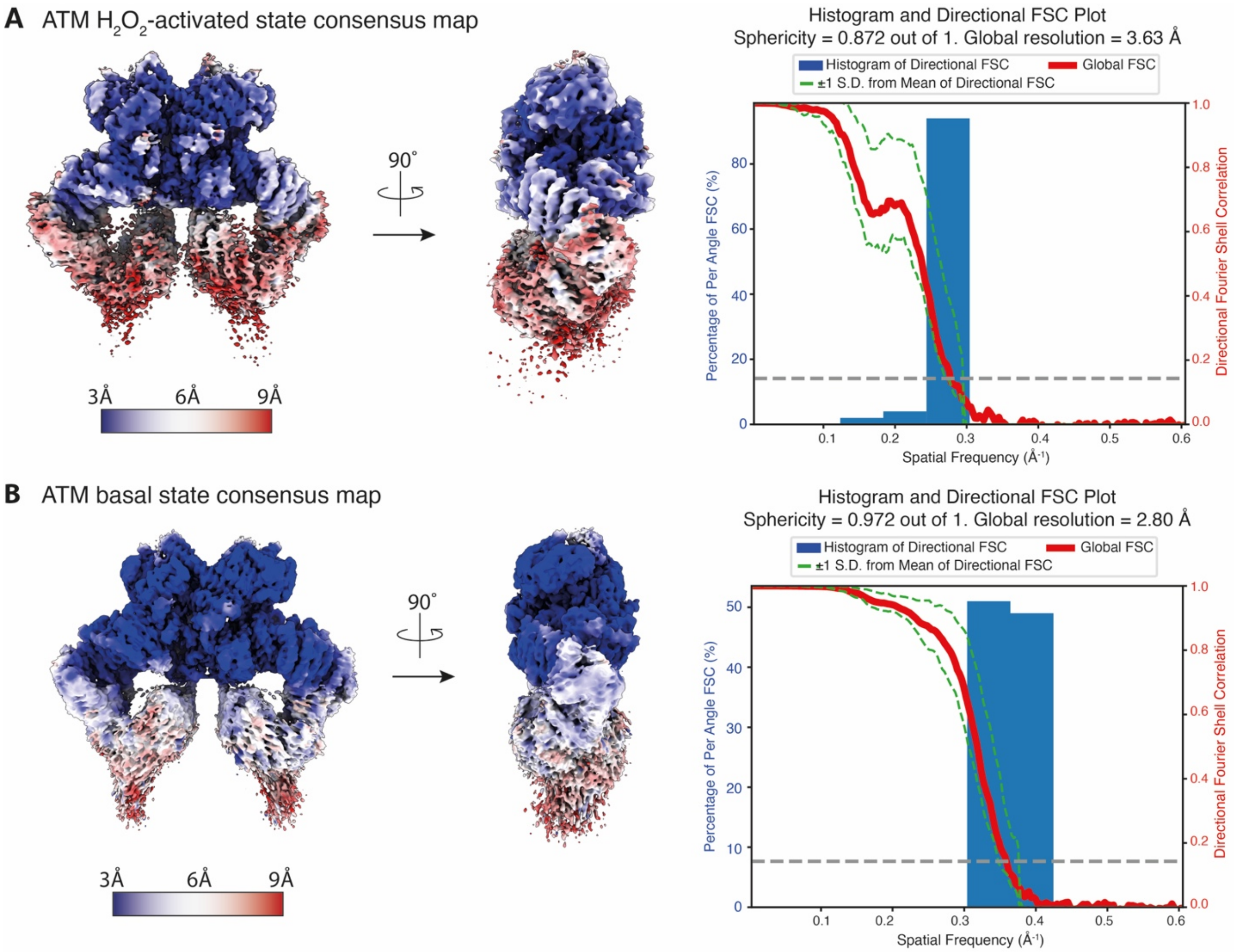

Fig. S6. Cryo-EM map quality metrics for the ATM H_2O_2-activated state and basal state dimer consensus maps. Front and side views of unsharpened EM density maps (left) for (**A**) ATM H_2O_2-activated state and (**B**) basal state colored by local resolution, estimated using cryoSPARC v4. Directional FSC plots and histograms for the final reconstructions were calculated using 3DFSC (right).

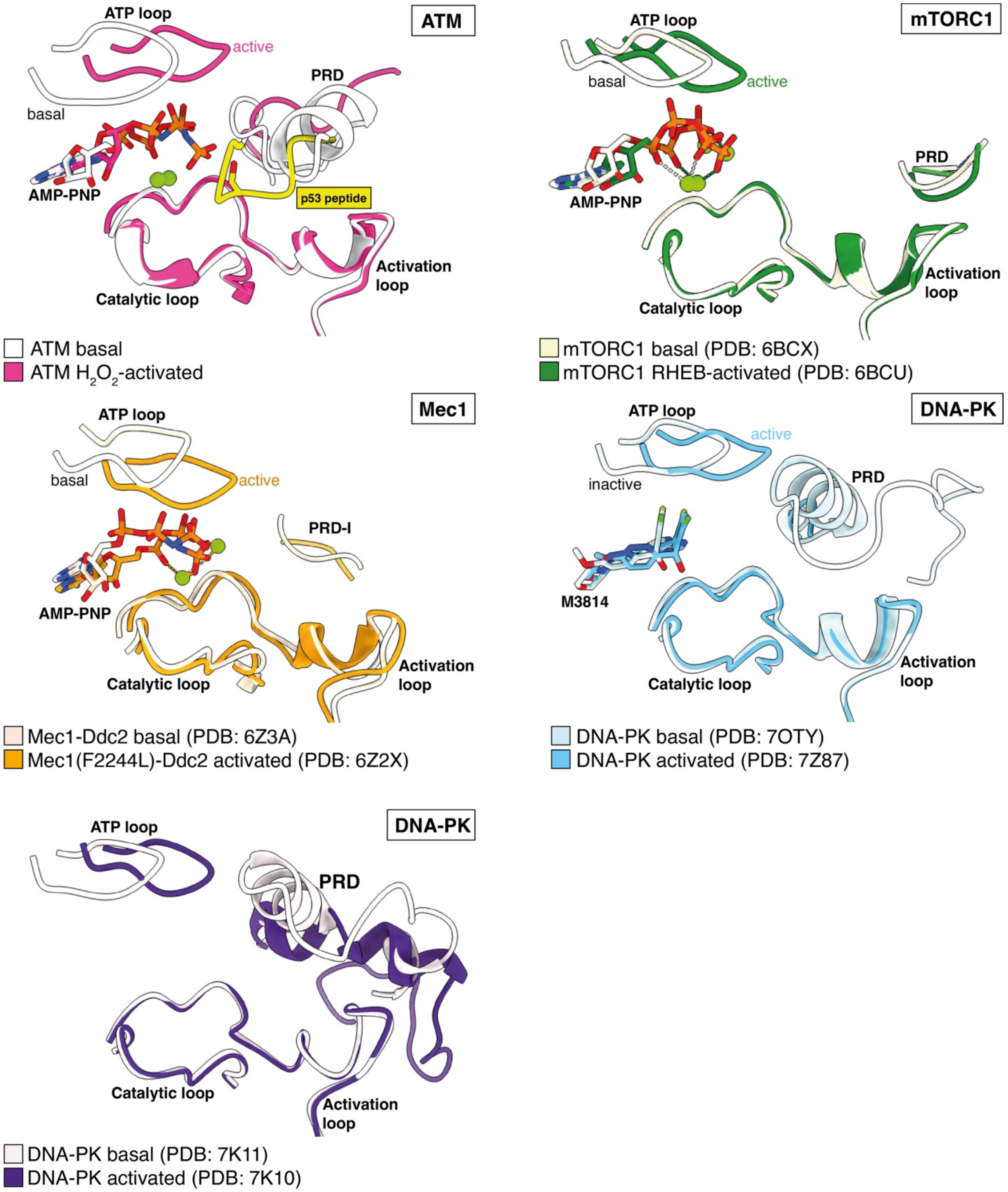

Fig. S7. ATP loop shifts are observed for the activated states of different PIKK family members. Models for basal and activated states of PIKK enzymes are aligned on the catalytic and activation loops in the kinase C-lobes. The activated states show a shift in the ATP loop relative to the basal state.

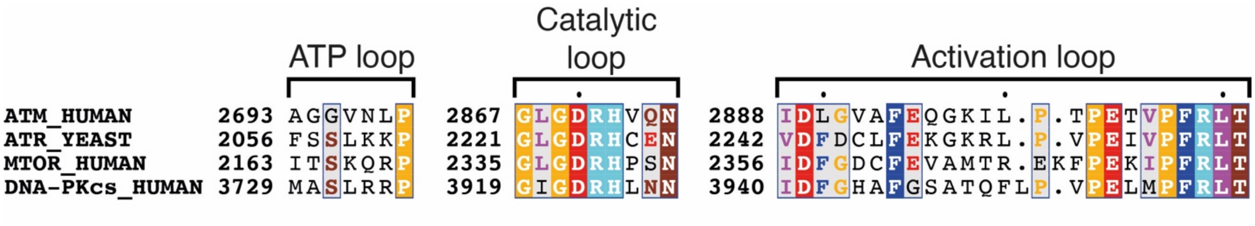

Fig. S8. Structure-based multiple sequence alignment of PIKKs. *H. sapiens* ATM, *S. cerevisiae* ATR (Mec1), *H. sapiens* mTOR, and *H. sapiens* DNA-PKcs sequences were aligned for the ATP loop, catalytic loop, and activation loop. The pairwise structure alignment tool from the PDB was first used on 7SIC (human ATM) paired with either 6Z2X (yeast ATR), 6BCX (human mTOR), or 7K11 (human DNA-PKcs). Sequence alignment was then performed for each pair for more extensive sequence coverage (to cover regions not included in PDB models) using MAFFT version 7 *(62)*. Uniprot sequences Q13315 (human ATM), P38111 (yeast ATR), P42345 (human mTOR), and P78527 (human DNA-PKcs) were used for the sequence inputs, and the pairwise structure alignment fasta file was used for additional constraints. Manual adjustments were then made in JalView (version 2.11.2.6) *(63)* to align the kinase domains. ESPript 3.0 *(64)*.

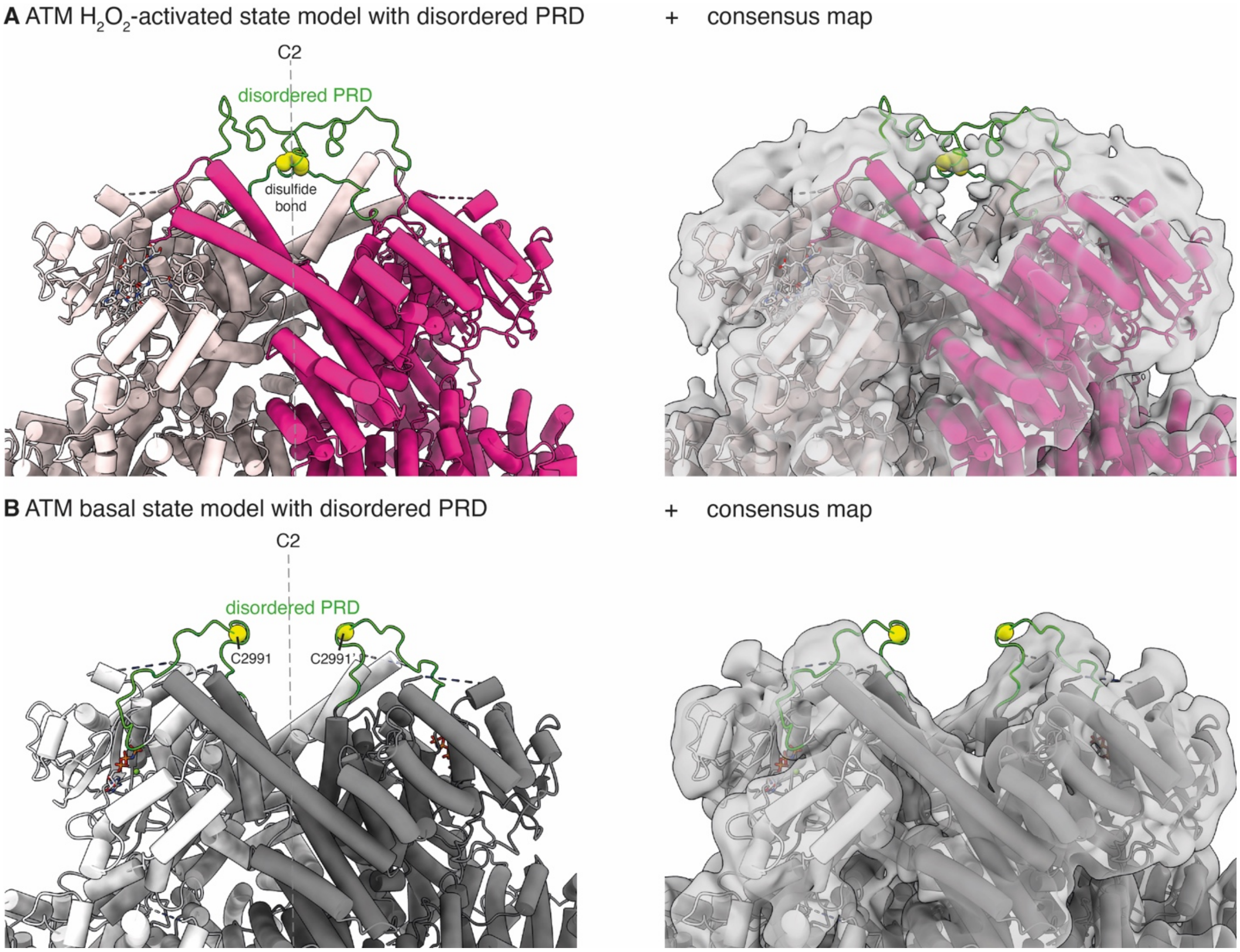

Fig. S9. Modelling of the disordered PRD regions into the ATM dimeric C-terminal regions. (**A**) A model for the H_2O_2-activated state with the addition of a plausible model for the disordered PRD region (left) and the 7 Å lowpass filtered consensus map superimposed on the model (right). (**B**) Same as in (**A**), but for the ATM basal state. The disordered PRD regions that cannot be modelled into high-resolution density of the locally refined dimeric C-terminal region maps are colored green and account for the remaining residues within the PRD. C2991 residues are indicated by yellow spheres.

A

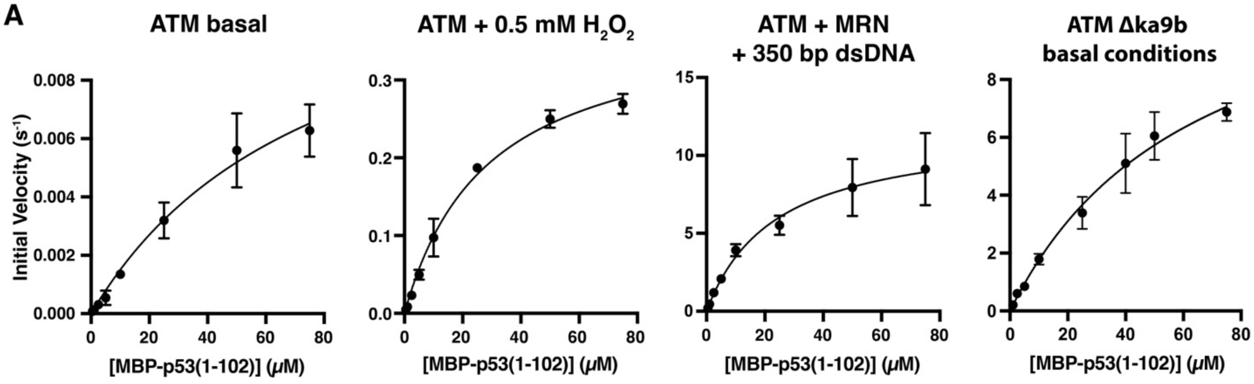

ATM basal	ATM + 0.5 mM H_2O_2	ATM + MRN + 350 bp dsDNA	ATM Δka9b basal conditions
k_{cat} = 0.015 ± 0.002 s^{-1}	k_{cat} = 0.38 ± 0.02 s^{-1}	k_{cat} = 12 ± 1 s^{-1}	k_{cat} = 13 ± 2 s^{-1}
K_M = 80 ± 24 μM	K_M = 29 ± 3 μM	K_M = 24 ± 6 μM	K_M = 65 ± 15 μM

	Initial velocity (s^{-1})											
	Basal			ATM + H_2O_2			ATM + MRN + 350 bp dsDNA			ATM Δkα9b basal conditions		
[MBP-p53[102]] (μM)	1	2	3	1	2	3	1	2	3	1	2	3
0.5	0.00008	0.00008	0.00013	0.00530	0.00520	0.00410	0.21000	0.22000	0.20000	0.23000	0.19000	0.20000
1	0.00014	0.00015	0.00010	0.00720	0.00880	0.00910	0.46000	0.48000	0.43000	0.60000	0.57000	0.64000
2.5	0.00037	0.00038	0.00023	0.01900	0.03000	0.02000	1.20000	1.33000	1.04000	0.95000	0.70000	0.89000
5	0.00076	0.00072	0.00045	0.04900	0.04300	0.05600	1.89000	2.11000	2.23000	1.82000	1.59000	1.96000
10	0.00134	0.00151	0.00138	0.12500	0.08700	0.08000	3.56000	4.33000	3.87000	4.00000	2.90000	3.28000
25	0.00338	0.00398	0.00257	0.18800	0.18700	0.18600	5.17000	6.22000	5.16000	6.15000	4.10000	5.06000
50	0.00577	0.00728	0.00500	0.24000	0.26200	0.24700	7.51000	9.95000	6.36000	7.00000	5.68000	5.47000
75	0.00678	0.00701	0.00633	0.27700	0.27700	0.25500	7.98000	11.79000	7.60000	7.00000	6.53000	7.10000

B Δkα9b(2963-2972)

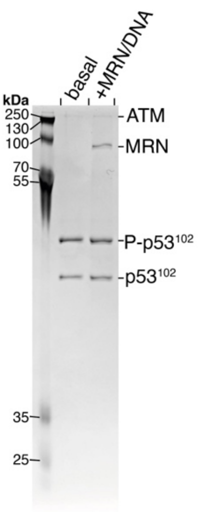

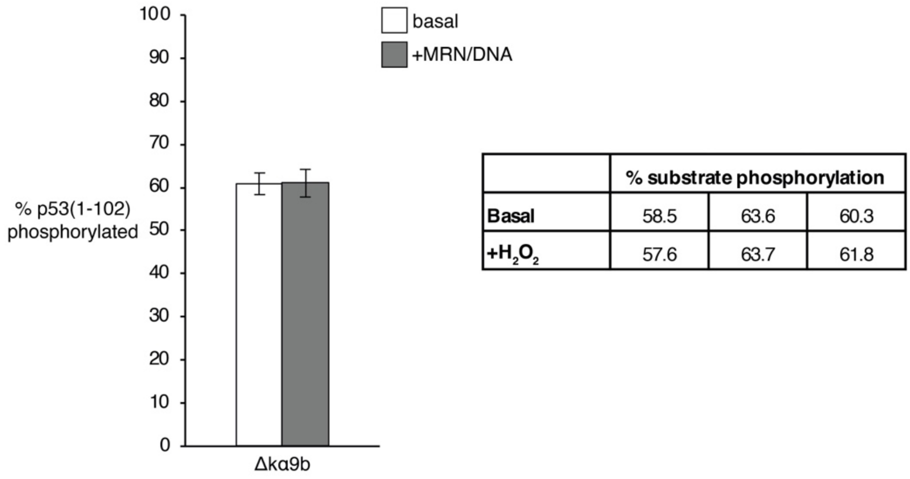

	% substrate phosphorylation		
**Basal**	58.5	63.6	60.3
**+H_2O_2**	57.6	63.7	61.8

Fig. S10. Measuring the kinase activity of ATM under different activating conditions.
(**A**) Michaelis-Menten plots and kinetic parameters were determined for ATM basal, H_2O_2-activating, and MRN/DNA-activating conditions using Prism 9. Concentrations: 0.5 µM ATM (basal condition), 50 nM ATM and 0.5 mM H_2O_2 (H_2O_2-activating condition), 10 nM ATM, 250 nM MRN and 10 nM 350 bp dsDNA (MRN/DNA-activating condition), 10 nM ATM(Δ2963-2972) (ATM Δkα9b basal condition). All samples contained varying concentrations of MBP-

p53^{102}, 1 mM ATP and 5 mM $MgCl_2$. Samples were incubated at 30 ˚C. For each MBP-p53^{102} concentration, incubation time was adjusted to fall within the linear range of kinase activity. Reactions were stopped with LDS sample buffer. Three biological replicates were used. Data values are displayed in a table below the graph. (**B**) Phos-tag gel comparing Δkα9b (2963-2972) kinase activity for basal versus MRN/DNA activating conditions (left). Concentrations (where used): 25 nM ATM, 250 nM MRN and 10 nM 350 bp dsDNA, 5 μM MBP-p53(1-102) (p53^{102}), 1 mM ATP and 5 mM $MgCl_2$. Samples were incubated at 30 ˚C for 2 min before being quenched with LDS sample buffer. Quantification of the Coomassie-stained Phos-tag gel bands to determine % p53(1-102) substrate phosphorylation using data from three biological replicates (right). Data values are displayed in a table to the right.

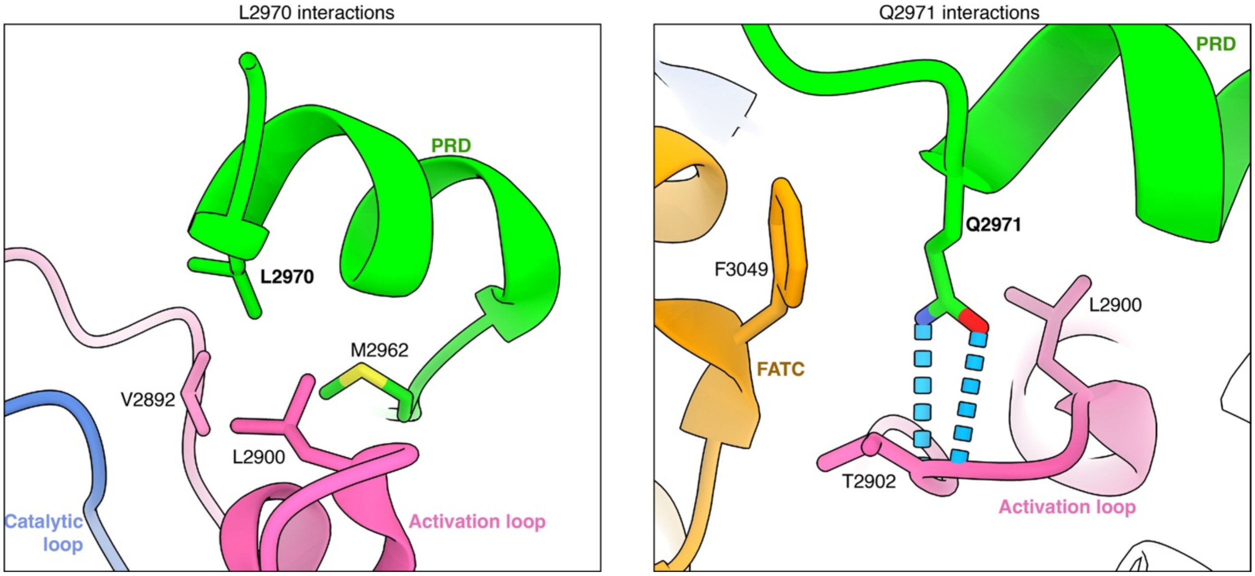

Fig. S11. Interactions of PRD residues L2970 and Q2971 with surrounding residues in basal state ATM. L2970A and Q2971A mutants were generated to destabilize the PRD from the substrate binding site. The interactions of the wild-type L2970 (left) and Q2971 (right) with the surrounding ATM residues are shown using PDB: 7SIC. L2970 makes hydrophobic interactions with residues V2892, L2900 (in the activation loop) and M2962 (in the PRD). Q2971 makes hydrogen bonds with the peptide backbone of T2902 (in the activation loop) and hydrophobic interactions with L2900 (in the activation loop) and F3049 (in FATC).

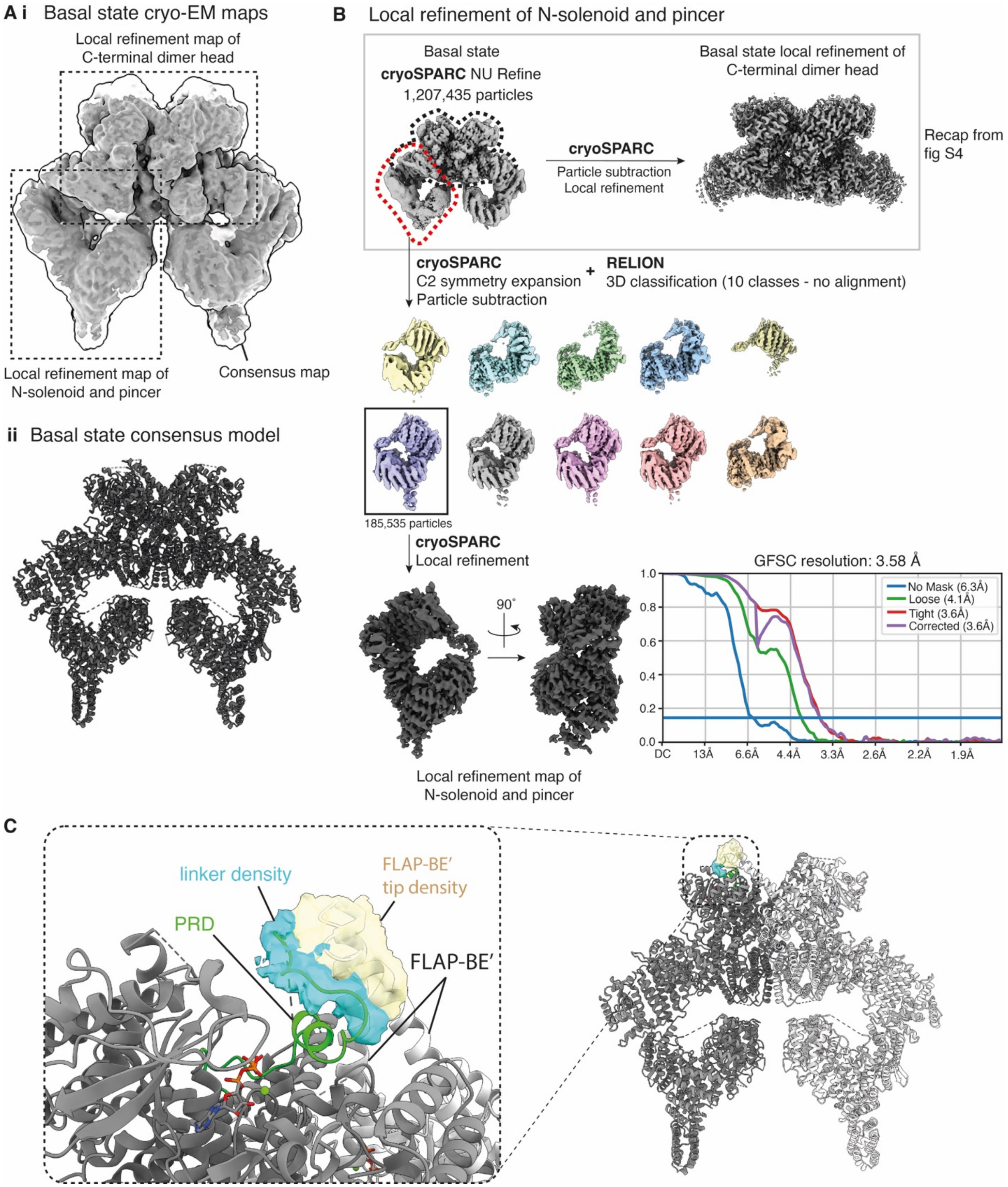

Fig. S12. ATM basal state cryo-EM maps. (**A**) (i) Local refinement maps for the N-terminus (two copies) and dimeric C-terminal region are docked into the whole dimer consensus map. (ii) The ATM basal state whole dimer model. (**B**) Processing steps for local refinement of the N-solenoid and pincer region. (**C**) Close-up view of the additional PRD linker density and FLAP-BE' tip density in the basal state map. The contact of these densities indicates potential interactions between PRD linker and FLAP-BE'.

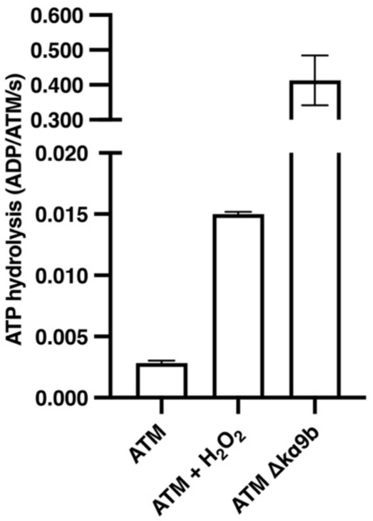

	ATP hydrolysis (ADP/ATM/s)		
**Sample**	1	2	3
**ATM**	0.003	0.003	0.003
**ATM + H_2O_2**	0.015	0.015	0.015
**ATM Δkα9b**	0.342	0.484	0.413

Fig. S13. ATP hydrolysis by ATM, using ADP-Glo kinase assay. ATP hydrolysis rates were measured for samples containing ATM without activator (basal), ATM with H_2O_2 and ATM Δkα9b mutant. Data were obtained from three biological replicates. Averages are plotted with error bars showing standard deviations. Data values are displayed in a table below the graph.

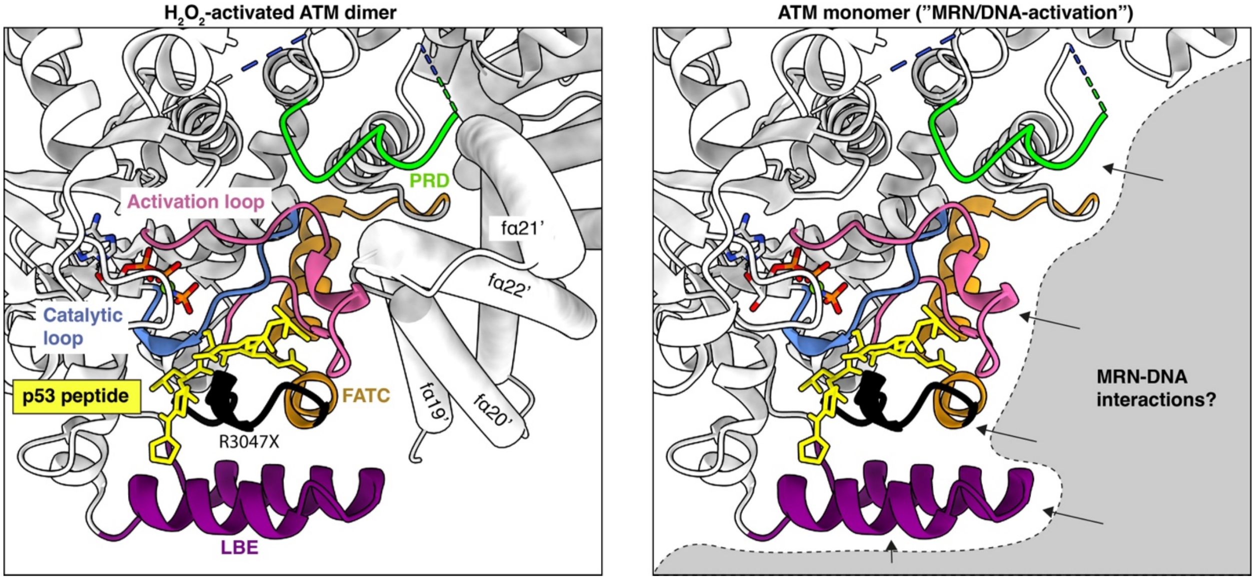

Fig. S14. Comparing the effects of R3047X on H_2O_2-activated ATM versus MRN/DNA-activated ATM. Mutation R3047X deletes the last ten residues in FATC (highlighted in black) that interact with the activation loop, catalytic loop, LBE and p53 peptide, as shown in the H_2O_2-activated state (left) where one protomer is represented with ribbons and the other protomer is represented with cylinders. Deletion of these residues in the H_2O_2-activated ATM dimer could destabilize the activation loop, catalytic loop and LBE, preventing an optimal conformation in the active site for catalysis and p53 substrate binding. ATM is hypothesized to form a monomer in the MRN/DNA activating condition, and so a single protomer derived from the H_2O_2-activated structure is used to represent this (right). MRN/DNA could potentially form additional stabilizing interactions with the LBE and activation loop that could not be provided by the other ATM protomer in the H_2O_2-activated ATM dimer.

	ATM(Q2971A) dimer in complex with AMP-PNP (EMDB-17267) (PDB 8OXP)	ATM(Q2971A) dimer activated by oxidative stress in complex with Mg AMP-PNP and p53 peptide (EMDB-17265) (PDB 8OXM)	ATM(Q2971A) dimeric C-terminal region in complex with Mg AMP-PNP (EMDB-17268) (PDB 8OXQ)	ATM(Q2971A) dimeric C-terminal region activated by oxidative stress in complex with Mg AMP-PNP and p53 peptide (EMDB-17266) (PDB 8OXO)
**Data collection and processing**				
Magnification	105,000	105,000	105,000	105,000
Voltage (kV)	300	300	300	300
Electron exposure (e–/Å^2)	34-45	34-45	34-45	34-45
Defocus range (µm)	-1.2 to -3.0	-1.2 to -3.0	-1.2 to -3.0	-1.2 to -3.0
Pixel size (Å)	0.826	0.826	0.826	0.826
Symmetry imposed	C2	C2	C2	C2
Initial particle images (no.)	4,529,936	4,529,936	4,529,936	4,529,936
Final particle images (no.)	1,207,435	30,707	1,207,435	30,707
Map resolution (Å)	2.6	3.3	2.5	3.0
FSC threshold	1.43	1.43	1.43	1.43
Map resolution range (Å)	2.6-3.2	3.2-4.3	2.5-2.9	3.0-3.6
**Refinement**				
Initial model used (PDB code)	PDB 7SIC	PDB 7SIC	PDB 7SIC	PDB 7SIC
Model resolution (Å)	3.1	4.0	2.8	3.5
FSC threshold	0.5	0.5	0.5	0.5
Model resolution range (Å)	2.6-3.2	3.2-4.3	2.5-2.9	3.0-3.6
Map sharpening *B* factor (Å^2)	-75	-65.9	-75	-74.3
Model composition				
Non-hydrogen atoms	44,460	44,174	25,078	23,986
Protein residues	5,544	5,510	3,100	2,966
Ligands	2 ANP<br>2 Mg^{2+}<br>2 Zn^{2+}	2 ANP<br>2 Mg^{2+}<br>2 Zn^{2+}	2 ANP<br>2 Mg^{2+}<br>2 Zn^{2+}	2 ANP<br>2 Mg^{2+}<br>2 Zn^{2+}
B factors (Å^2)				
Protein	317.15	244.42	142.53	168.79
Ligand	145.46	132.69	172.24	176.03
R.m.s. deviations				
Bond lengths (Å)	0.002	0.002	0.001	0.003
Bond angles (°)	0.467	0.474	0.426	0.493
Validation				
MolProbity score	1.34	1.47	1.28	1.43
Clashscore	6.15	8.21	5.30	7.05
Poor rotamers (%)	0.00	0.00	0.00	0.00
Ramachandran plot				
Favored (%)	98.21	97.92	98.37	97.82
Allowed (%)	1.79	2.08	1.63	2.18
Disallowed (%)	0.00	0.00	0.00	0.00

Table S1. Cryo-EM data collection, refinement, and validation statistics.

Basal State		H_2O_2-activated state	
**Feature in protomer A**	**Feature in protomer B**	**Feature in protomer A**	**Feature in protomer B**
kα10/FATC loop<br>(3022)	FLAP-BE’<br>(2400)	kα10 loop/FATC, FATC<br>(3022-3024)	FLAP-BE’<br>(2400, 2404, 2455)
LBE<br>(2810, 2811, 2813)	FLAP-BE’<br>(2443, 2444, 2447, 2450, 2451, 2454)	LBE<br>(None)	FLAP-BE’<br>(None)
Activation Loop<br>(2898, 2899, 2901, 2902)	FLAP-BE’<br>(2400, 2408, 2409, 2412, 2413, 2416, 2444,2447, 2451)	Activation Loop<br>(2898, 2899, 2900)	FLAP-BE’<br>(2440, 2443, 2444, 2447)
PRD<br>(2962, 2964, 2968, 2973)	FLAP-BE’<br>(2416, 2420, 2440, 2443, 2444)	PRD<br>(2959, 2960, 2962, 2963)	FLAP-BE’<br>(2427, 2432, 2435, 2436, 2437, 2440)
kα10<br>(3005, 3008, 3018)	FLAP-BE’<br>(2408, 2412)	kα10<br>(3002, 3004, 3005, 3007, 3008, 3011)	FLAP-BE’<br>(2413, 2416, 2419, 2437, 2440, 2444, 2448)
FATC<br>(3023, 3025)	kα10’<br>(3018)	FATC<br>(None)	kα10’<br>(None)
FATC<br>(3033, 3034, 3037, 3041)	fα19’/fα20’ loop<br>(2348, 2349, 2350, 2352)	FATC<br>(3024-3027, 3030, 3033, 3034, 3037)	fα19’/fα20’ loop<br>(2349-2352)

Table S2. Features and residues involved at the upper dimeric interface between ATM protomers. Features and residues that are involved at the upper dimeric interface are shown and juxtaposed for the ATM basal state (left) and ATM H_2O_2-activated state (right). Residues that have a buried residue area of at least 15 Å are considered to be interface residues and are listed in brackets below the associated feature.

Plasmid Number	Vector	Protein
AHp16	pDEST12.2-OriP	Flag-FKBP1A-9AA linker-HsATM
AHp28	pDEST12.2-OriP	Flag-FKBP1A-9AA linker-HsATM D2870A/N2875K
AHp42	pDEST12.2-OriP	Flag-FKBP1A-9AA linker-HsATM C2991S
AHp45	pDEST12.2-OriP	Flag-FKBP1A-9AA linker-HsATM C2991L
AHp47	pDEST12.2-OriP	Flag-FKBP1A-9AA linker-HsATM Q2971A
AHp57	pDEST12.2-OriP	Flag-FKBP1A-9AA linker-HsATM L2970A
AHp72	pDEST12.2-OriP	Flag-FKBP1A-9AA linker-HsATM Δ2973-2988 (Δlinker)
AHp74	pDEST12.2-OriP	Flag-FKBP1A-9AA linker-HsATM Δ2963-2972 (Δkα9b)
AHp77	pDEST12.2-OriP	Flag-FKBP1A-9AA linker-HsATM 2975-2988 to GSGGSGGSGGSGGS (linker GSG_n)
AHp79	pDEST12.2-OriP	Flag-FKBP1A-9AA linker-HsATM 2968-LYLQ-2971 to AAAA
AHp63	pFastBac	HsRad50-TEV-2xStrep
AHp64	pFastBac Dual	HsMre11 (untagged) and HsNbs1-TEV-2xStrep
AHp26	pOPTHM(TEV)	6xHis-MBP-TEV-p53(1-102)
AHp32	pOPTHM(TEV)	6xHis-MBP-TEV-NRF1 (full-length)
AHp46	pOPTHM(TEV)	6xHis-MBP-TEV-CHK2(1-107)

Table S3. Plasmids used for protein expression.

Movie S1. The ATM dimer is twisted in the ATM H_2O_2-activated state compared to the basal state. ATM protomer rotation is observed when morphing between the basal state model and H_2O_2-activated state model. Models are aligned on the right-hand side protomer (gray or dark pink) to observe changes occurring in the left-hand side protomer (white or light pink).

Movie S2. An additional bridge density is observed between the two ATM protomers in the H_2O_2-activated state consensus map. An additional bridging density is observed in the 7 Å lowpass-filtered H_2O_2-activated state ATM consensus map. The grey map (consensus map) has its density threshold lowered whilst the pink map (the high resolution 3 Å sharpened C terminus dimer map) remains at a higher density threshold and is used as a reference to show the relative position of the bridge between ATM protomers.

Movie S3. The upper dimeric interface changes in the H_2O_2-activated state compared to the basal state. Dimeric interface interactions change going from the basal state to the H_2O_2-activated state of ATM. The movie begins with the ATM dimeric C-terminal region, then focuses in on the dimeric interface on one side of the dimer. Elements involved at the dimeric interface are colored and then morphing occurs from the basal state to the H_2O_2-activated state.

Movie S4. The kinase N-lobe rotates relative to the C-lobe in the H_2O_2-activated state compared to the basal state. A morph movie demonstrating the rotation of the kinase N-lobe (white or light pink) relative to the C-lobe (gray or deep pink) going from the basal state model (white and gray) to the H_2O_2-activated state model (light and deep pink). The models are aligned on the kinase C-lobes to focus on motion in the N-lobe.

Movie S5. Remodeling of the kinase domain upon H_2O_2-mediated activation. A morph movie of the kinase domain going from H_2O_2-activated ATM with p53 substrate bound to the basal state (PDB: 7SIC). Similarly, the bound ATP analogue of the activated state was morphed onto the ATP analogue of the basal state. Only the kinase domain and its substrates are shown. The p53 substrate peptide bound in the active site is shown as magenta sticks. Side chains are shown for the C-lobe residues that make up the pocket into which the p53 Q16 (position +1) side chain inserts. In the activated state, helix kα9b of the PRD is mostly disordered and not seen in the structure. For the movie, helix kα9b, which is ordered and wedged between the N- and C-lobes in the basal state, is shown lifting out of the active site upon activation and assuming an arbitrary position, while the p53 substrate peptide slides into the emptied active site. The side chain for Q2971 in helix kα9b is illustrated as sticks to emphasize that in the basal state Q2971 occupies the same pocket as Q16 of the substrate peptide in the activated state. Upon activation, the N-lobe of the kinase domain (red) rotates relative to the C-lobe (yellow). This causes the bound ATP analogue (cyan, AMPPNP) to change its conformation, altering interactions with the bound Mg^{2+} (large cyan sphere), and changing ATP contacts with the N-lobe. Once the p53 substrate is in place, the γ-phosphate of the ATP analogue (light blue sphere) forms a close interaction with S15 of the substrate peptide.

REFERENCES AND NOTES

1. C. Rothblum-Oviatt, J. Wright, M. A. Lefton-Greif, S. A. McGrath-Morrow, T. O. Crawford, H. M. Lederman, Ataxia telangiectasia: A review. *Orphanet J. Rare Dis.* **11**, 159 (2016).

2. N. Takao, Y. Li, K. Yamamoto, Protective roles for ATM in cellular response to oxidative stress. *FEBS Lett.* **472**, 133–136 (2000).

3. J. Reichenbach, R. Schubert, D. Schindler, K. Muller, H. Bohles, S. Zielen, Elevated oxidative stress in patients with ataxia telangiectasia. *Antioxid. Redox Signal.* **4**, 465–469 (2002).

4. G. Rotman, Y. Shiloh, The ATM gene and protein: Possible roles in genome surveillance, checkpoint controls and cellular defence against oxidative stress. *Cancer Surv.* **29**, 285–304 (1997).

5. G. Rotman, Y. Shiloh, Hypothesis: Ataxia-telangiectasia: Is ATM a sensor of oxidative damage and stress? *Bioessays* **19**, 911–917 (1997).

6. M. Ambrose, J. V. Goldstine, R. A. Gatti, Intrinsic mitochondrial dysfunction in ATM-deficient lymphoblastoid cells. *Hum. Mol. Genet.* **16**, 2154–2164 (2007).

7. H. M. Chow, A. Cheng, X. Song, M. R. Swerdel, R. P. Hart, K. Herrup, ATM is activated by ATP depletion and modulates mitochondrial function through NRF1. *J. Cell Biol.* **218**, 909–928 (2019).

8. J. H. Lee, M. R. Mand, C. H. Kao, Y. Zhou, S. W. Ryu, A. L. Richards, J. J. Coon, T. T. Paull, ATM directs DNA damage responses and proteostasis via genetically separable pathways. *Sci. Signal.* **11**, (2018).

9. Y. A. Valentin-Vega, K. H. Maclean, J. Tait-Mulder, S. Milasta, M. Steeves, F. C. Dorsey, J. L. Cleveland, D. R. Green, M. B. Kastan, Mitochondrial dysfunction in ataxia-telangiectasia. *Blood* **119**, 1490–1500 (2012).

10. Z. Guo, S. Kozlov, M. F. Lavin, M. D. Person, T. T. Paull, ATM activation by oxidative stress. *Science* **330**, 517–521 (2010).

11. R. E. Shackelford, C. L. Innes, S. O. Sieber, A. N. Heinloth, S. A. Leadon, R. S. Paules, The Ataxia telangiectasia gene product is required for oxidative stress-induced G1 and G2 checkpoint function in human fibroblasts. *J. Biol. Chem.* **276**, 21951–21959 (2001).

12. E. F. Fang, H. Kassahun, D. L. Croteau, M. Scheibye-Knudsen, K. Marosi, H. Lu, R. A. Shamanna, S. Kalyanasundaram, R. C. Bollineni, M. A. Wilson, W. B. Iser, B. N. Wollman, M. Morevati, J. Li, J. S. Kerr, Q. Lu, T. B. Waltz, J. Tian, D. A. Sinclair, M. P. Mattson, H. Nilsen, V. A. Bohr, NAD(+) replenishment improves lifespan and healthspan in ataxia telangiectasia models via mitophagy and DNA repair. *Cell Metab.* **24**, 566–581 (2016).

13. J. H. Lee, T. T. Paull, Mitochondria at the crossroads of ATM-mediated stress signaling and regulation of reactive oxygen species. *Redox Biol.* **32**, 101511 (2020).

14. C. Cosentino, D. Grieco, V. Costanzo, ATM activates the pentose phosphate pathway promoting anti-oxidant defence and DNA repair. *EMBO J.* **30**, 546–555 (2011).

15. C. R. Reczek, N. S. Chandel, The two faces of reactive oxygen species in cancer. *Annu. Rev. Cancer Biol.* **1**, 79–98 (2017).

16. D. Baretic, R. L. Williams, PIKKs — the solenoid nest where partners and kinases meet. *Curr. Opin. Struct. Biol.* **29**, 134–142 (2014).

17. J. Perry, N. Kleckner, The ATRs, ATMs, and TORs are giant HEAT repeat proteins. *Cell* **112**, 151–155 (2003).

18. D. Baretic, H. K. Pollard, D. I. Fisher, C. M. Johnson, B. Santhanam, C. M. Truman, T. Kouba, A. R. Fersht, C. Phillips, R. L. Williams, Structures of closed and open conformations of dimeric human ATM. *Sci. Adv.* **3**, e1700933 (2017).

19. W. C. Lau, Y. Li, Z. Liu, Y. Gao, Q. Zhang, M. S. Huen, Structure of the human dimeric ATM kinase. *Cell Cycle* **15**, 1117–1124 (2016).

20. K. Stakyte, M. Rotheneder, K. Lammens, J. D. Bartho, U. Gradler, T. Fuchss, U. Pehl, A. Alt, E. van de Logt, K. P. Hopfner, Molecular basis of human ATM kinase inhibition. *Nat. Struct. Mol. Biol.* **28**, 789–798 (2021).

21. C. Warren, N. P. Pavletich, Structure of the human ATM kinase and mechanism of Nbs1 binding. *eLife* **11**, e74218 (2022).

22. J. Xiao, M. Liu, Y. Qi, Y. Chaban, C. Gao, B. Pan, Y. Tian, Z. Yu, J. Li, P. Zhang, Y. Xu, Structural insights into the activation of ATM kinase. *Cell Res.* **29**, 683–685 (2019).

23. X. Wang, H. Chu, M. Lv, Z. Zhang, S. Qiu, H. Liu, X. Shen, W. Wang, G. Cai, Structure of the intact ATM/Tel1 kinase. *Nat. Commun.* **7**, 11655 (2016).

24. M. Jansma, C. Linke-Winnebeck, S. Eustermann, K. Lammens, D. Kostrewa, K. Stakyte, C. Litz, B. Kessler, K. P. Hopfner, Near-complete structure and model of Tel1ATM from chaetomium thermophilum reveals a robust autoinhibited ATP state. *Structure* **28**, 83–95.e5 (2020).

25. M. Sawicka, P. H. Wanrooij, V. C. Darbari, E. Tannous, S. Hailemariam, D. Bose, A. V. Makarova, P. M. Burgers, X. Zhang, The dimeric architecture of checkpoint kinases Mec1ATR and Tel1ATM reveal a common structural organization. *J. Biol. Chem.* **291**, 13436–13447 (2016).

26. J. Xin, Z. Xu, X. Wang, Y. Tian, Z. Zhang, G. Cai, Structural basis of allosteric regulation of Tel1/ATM kinase. *Cell Res.* **29**, 655–665 (2019).

27. L. A. Yates, R. M. Williams, S. Hailemariam, R. Ayala, P. Burgers, X. Zhang, Cryo-EM structure of nucleotide-bound Tel1(ATM) unravels the molecular basis of inhibition and structural rationale for disease-associated mutations. *Structure* **28**, 96–104.e3 (2020).

28. H. Yang, X. Jiang, B. Li, H. J. Yang, M. Miller, A. Yang, A. Dhar, N. P. Pavletich, Mechanisms of mTORC1 activation by RHEB and inhibition by PRAS40. *Nature* **552**, 368–373 (2017).

29. C. J. Bakkenist, M. B. Kastan, DNA damage activates ATM through intermolecular autophosphorylation and dimer dissociation. *Nature* **421**, 499–506 (2003).

30. J. H. Lee, T. T. Paull, Direct activation of the ATM protein kinase by the Mre11/Rad50/Nbs1 complex. *Science* **304**, 93–96 (2004).

31. L. O'Donoghue, A. Smolenski, Analysis of protein phosphorylation using Phos-tag gels. *J. Proteomics* **259**, 104558 (2022).

32. J. H. Lee, T. T. Paull, ATM activation by DNA double-strand breaks through the Mre11-Rad50-Nbs1 complex. *Science* **308**, 551–554 (2005).

33. C. Cirotti, S. Rizza, P. Giglio, N. Poerio, M. F. Allega, G. Claps, C. Pecorari, J. H. Lee, B. Benassi, D. Barilà, C. Robert, J. S. Stamler, F. Cecconi, M. Fraziano, T. T. Paull, G. Filomeni, Redox activation of ATM enhances GSNOR translation to sustain mitophagy and tolerance to oxidative stress. *EMBO Rep.* **22**, e50500 (2021).

34. L. M. Langer, Y. Gat, F. Bonneau, E. Conti, Structure of substrate-bound SMG1-8-9 kinase complex reveals molecular basis for phosphorylation specificity. *eLife* **9**, e57127 (2020).

35. S. T. Kim, D. S. Lim, C. E. Canman, M. B. Kastan, Substrate specificities and identification of putative substrates of ATM kinase family members. *J. Biol. Chem.* **274**, 37538–37543 (1999).

36. J. L. Johnson, T. M. Yaron, E. M. Huntsman, A. Kerelsky, J. Song, A. Regev, T. Y. Lin, K. Liberatore, D. M. Cizin, B. M. Cohen, N. Vasan, Y. Ma, K. Krismer, J. T. Robles, B. van de Kooij, A. E. van Vlimmeren, N. Andrée-Busch, N. F. Käufer, M. V. Dorovkov, A. G. Ryazanov, Y. Takagi, E. R. Kastenhuber, M. D. Goncalves, B. D. Hopkins, O. Elemento, D. J. Taatjes, A. Maucuer, A. Yamashita, A. Degterev, M. Uduman, J. Lu, S. D. Landry, B. Zhang, I. Cossentino, R. Linding, J. Blenis, P. V. Hornbeck, B. E. Turk, M. B. Yaffe, L. C. Cantley, An atlas of substrate specificities for the human serine/threonine kinome. *Nature* **613**, 759–766 (2023).

37. T. O'Neill, A. J. Dwyer, Y. Ziv, D. W. Chan, S. P. Lees-Miller, R. H. Abraham, J. H. Lai, D. Hill, Y. Shiloh, L. C. Cantley, G. A. Rathbun, Utilization of oriented peptide libraries to identify substrate motifs selected by ATM. *J. Biol. Chem.* **275**, 22719–22727 (2000).

38. E. A. Tannous, L. A. Yates, X. Zhang, P. M. Burgers, Mechanism of auto-inhibition and activation of Mec1(ATR) checkpoint kinase. *Nat. Struct. Mol. Biol.* **28**, 50–61 (2021).

39. S. Liang, T. L. Blundell, Human DNA-dependent protein kinase activation mechanism. *Nat. Struct. Mol. Biol.* **30**, 140–147 (2023).

40. X. Chen, X. Xu, Y. Chen, J. C. Cheung, H. Wang, J. Jiang, N. de Val, T. Fox, M. Gellert, W. Yang, Structure of an activated DNA-PK and its implications for NHEJ. *Mol. Cell* **81**, 801–810.e3 (2021).

41. A. Corcoran, T. G. Cotter, Redox regulation of protein kinases. *FEBS J.* **280**, 1944–1965 (2013).

42. T. H. Truong, K. S. Carroll, Redox regulation of protein kinases. *Crit. Rev. Biochem. Mol. Biol.* **48**, 332–356 (2013).

43. S. V. Kozlov, A. J. Waardenberg, K. Engholm-Keller, J. W. Arthur, M. E. Graham, M. Lavin, Reactive oxygen species (ROS)-activated ATM-dependent phosphorylation of cytoplasmic substrates identified by large-scale phosphoproteomics screen. *Mol. Cell. Proteomics* **15**, 1032–1047 (2016).

44. J. H. Lee, T. T. Paull, Cellular functions of the protein kinase ATM and their relevance to human disease. *Nat. Rev. Mol. Cell Biol.* **22**, 796–814 (2021).

45. K. Li, G. Bronk, J. Kondev, J. E. Haber, Yeast ATM and ATR kinases use different mechanisms to spread histone H2A phosphorylation around a DNA double-strand break. *Proc. Natl. Acad. Sci. U.S.A.* **117**, 21354–21363 (2020).

46. A. C. Joerger, M. D. Allen, A. R. Fersht, Crystal structure of a superstable mutant of human p53 core domain. Insights into the mechanism of rescuing oncogenic mutations. *J. Biol. Chem.* **279**, 1291–1296 (2004).

47. P. V. Nikolova, J. Henckel, D. P. Lane, A. R. Fersht, Semirational design of active tumor suppressor p53 DNA binding domain with enhanced stability. *Proc. Natl. Acad. Sci. U.S.A.* **95**, 14675–14680 (1998).

48. Z. Zhang, J. Yang, D. Barford, Recombinant expression and reconstitution of multiprotein complexes by the USER cloning method in the insect cell-baculovirus expression system. *Methods* **95**, 13–25 (2016).

49. J. Zivanov, T. Nakane, B. O. Forsberg, D. Kimanius, W. J. Hagen, E. Lindahl, S. H. Scheres, New tools for automated high-resolution cryo-EM structure determination in RELION-3. *eLife* **7**, e42166 (2018).

50. S. Q. Zheng, E. Palovcak, J. P. Armache, K. A. Verba, Y. Cheng, D. A. Agard, MotionCor2: Anisotropic correction of beam-induced motion for improved cryo-electron microscopy. *Nat. Methods* **14**, 331–332 (2017).

51. A. Rohou, N. Grigorieff, CTFFIND4: Fast and accurate defocus estimation from electron micrographs. *J. Struct. Biol.* **192**, 216–221 (2015).

52. T. Wagner, F. Merino, M. Stabrin, T. Moriya, C. Antoni, A. Apelbaum, P. Hagel, O. Sitsel, T. Raisch, D. Prumbaum, D. Quentin, D. Roderer, S. Tacke, B. Siebolds, E. Schubert, T. R. Shaikh, P. Lill, C. Gatsogiannis, S. Raunser, SPHIRE-crYOLO is a fast and accurate fully automated particle picker for cryo-EM. *Commun. Biol.* **2**, 218 (2019).

53. A. Punjani, J. L. Rubinstein, D. J. Fleet, M. A. Brubaker, cryoSPARC: Algorithms for rapid unsupervised cryo-EM structure determination. *Nat. Methods* **14**, 290–296 (2017).

54. E. F. Pettersen, T. D. Goddard, C. C. Huang, G. S. Couch, D. M. Greenblatt, E. C. Meng, T. E. Ferrin, UCSF Chimera—A visualization system for exploratory research and analysis. *J. Comput. Chem.* **25**, 1605–1612 (2004).

55. D. Asarnow, E. Palovcak, Y. Cheng, UCSF pyem v0.5. Zenodo. (2019).

56. A. Punjani, D. J. Fleet, 3D variability analysis: Resolving continuous flexibility and discrete heterogeneity from single particle cryo-EM. *J. Struct. Biol.* **213**, 107702 (2021).

57. J. Zivanov, T. Nakane, S. H. W. Scheres, A Bayesian approach to beam-induced motion correction in cryo-EM single-particle analysis. *IUCrJ* **6**, 5–17 (2019).

58. P. Emsley, B. Lohkamp, W. G. Scott, K. Cowtan, Features and development of Coot. *Acta Crystallogr. D Biol. Crystallogr.* **66**, 486–501 (2010).

59. P. D. Adams, P. V. Afonine, G. Bunkoczi, V. B. Chen, I. W. Davis, N. Echols, J. J. Headd, L. W. Hung, G. J. Kapral, R. W. Grosse-Kunstleve, A. J. McCoy, N. W. Moriarty, R. Oeffner, R. J. Read, D. C. Richardson, J. S. Richardson, T. C. Terwilliger, P. H. Zwart, PHENIX: A comprehensive python-based system for macromolecular structure solution. *Acta Crystallogr. D Biol. Crystallogr.* **66**, 213–221 (2010).

60. I. Hickson, Y. Zhao, C. J. Richardson, S. J. Green, N. M. Martin, A. I. Orr, P. M. Reaper, S. P. Jackson, N. J. Curtin, G. C. Smith, Identification and characterization of a novel and specific inhibitor of the ataxia-telangiectasia mutated kinase ATM. *Cancer Res.* **64**, 9152–9159 (2004).

61. Y. Z. Tan, P. R. Baldwin, J. H. Davis, J. R. Williamson, C. S. Potter, B. Carragher, D. Lyumkis, Addressing preferred specimen orientation in single-particle cryo-EM through tilting. *Nat. Methods* **14**, 793–796 (2017).

62. J. Rozewicki, S. Li, K. M. Amada, D. M. Standley, K. Katoh, MAFFT-DASH: Integrated protein sequence and structural alignment. *Nucleic Acids Res.* **47**, W5–w10 (2019).

63. A. M. Waterhouse, J. B. Procter, D. M. Martin, M. Clamp, G. J. Barton, Jalview Version 2—a multiple sequence alignment editor and analysis workbench. *Bioinformatics* **25**, 1189–1191 (2009).

64. X. Robert, P. Gouet, Deciphering key features in protein structures with the new ENDscript server. *Nucleic Acids Res.* **42**, W320–W324 (2014).
